# Non-Coding RNAs in Prediabetes Beyond Glycemic Classification: Tissue Regulatory Networks, Progression Risk, and Intervention-Associated Dynamics

**DOI:** 10.3390/biom16101472

**Published:** 2026-10-09

**Authors:** Zion Kim, Eun Yeong Ha, Jae-Hyung Park

**Affiliations:** 1 Medical Course, Keimyung University School of Medicine, Daegu 42601, Republic of Korea; zionkim1207@gmail.com; 2 Department of Internal Medicine, Keimyung University School of Medicine, Daegu 42601, Republic of Korea; 3 Department of Physiology, Keimyung University School of Medicine, Daegu 42601, Republic of Korea

**Keywords:** prediabetes, non-coding RNA, microRNA, long non-coding RNA, circular RNA, competing endogenous RNA, risk stratification, pharmacodynamic biomarkers, diabetes prevention program

## Abstract

Prediabetes can be readily identified using established glycemic tests, but these measures provide limited information about underlying molecular heterogeneity, future progression to type 2 diabetes mellitus (T2DM), or molecular responses to preventive interventions. This narrative review evaluates whether non-coding RNAs (ncRNAs) may provide complementary information beyond glycemic classification. We integrate evidence on microRNAs (miRNAs), long non-coding RNAs (lncRNAs), and circular RNAs (circRNAs), with brief consideration of emerging small ncRNA classes and epitranscriptomic regulation, emphasizing tissue-resolved regulatory crosstalk in skeletal muscle, liver, pancreatic β-cells, and adipose tissue while distinguishing direct human prediabetes evidence from mechanistic findings derived from related metabolic contexts. Circulating and extracellular-vesicle-associated ncRNAs are critically assessed according to diagnostic, prognostic, predictive, and pharmacodynamic functions. Particular attention is given to Diabetes Prevention Program analyses, in which baseline miRNA profiles were associated with incident T2DM and showed an exploratory treatment-by-biomarker interaction, while metformin exposure was associated with longitudinal changes in circulating miRNAs. Current evidence supports ncRNAs primarily as hypothesis-generating tools for mechanistic discovery and as candidate markers for progression risk and intervention-associated molecular dynamics, rather than replacements for established glycemic testing. Prospective validation is required to determine whether ncRNA profiling adds clinically meaningful information beyond conventional risk factors.

## 1. Introduction

Prediabetes is defined by glycemic values that are above the normal range but below the diagnostic thresholds for type 2 diabetes mellitus (T2DM) and represents a major high-risk state for subsequent diabetes development [1,2]. Its clinical importance extends beyond its high prevalence because metabolic deterioration begins before overt diabetes is established. Longitudinal studies have demonstrated progressive changes in insulin sensitivity, compensatory insulin secretion, and pancreatic β-cell function during the years preceding T2DM [3]. However, prediabetes is not a uniform or inevitably progressive condition. Some individuals progress rapidly to T2DM, whereas others remain metabolically stable or return to normoglycemia, either spontaneously or following intervention [2,4,5]. The Diabetes Prevention Program (DPP) and other large prevention trials further demonstrated that lifestyle modification and metformin can substantially modify this trajectory [6,7,8]. Prediabetes can therefore be viewed not only as an intermediate glycemic category but also as a biologically heterogeneous and potentially modifiable stage of metabolic disease.

**Figure 1:**
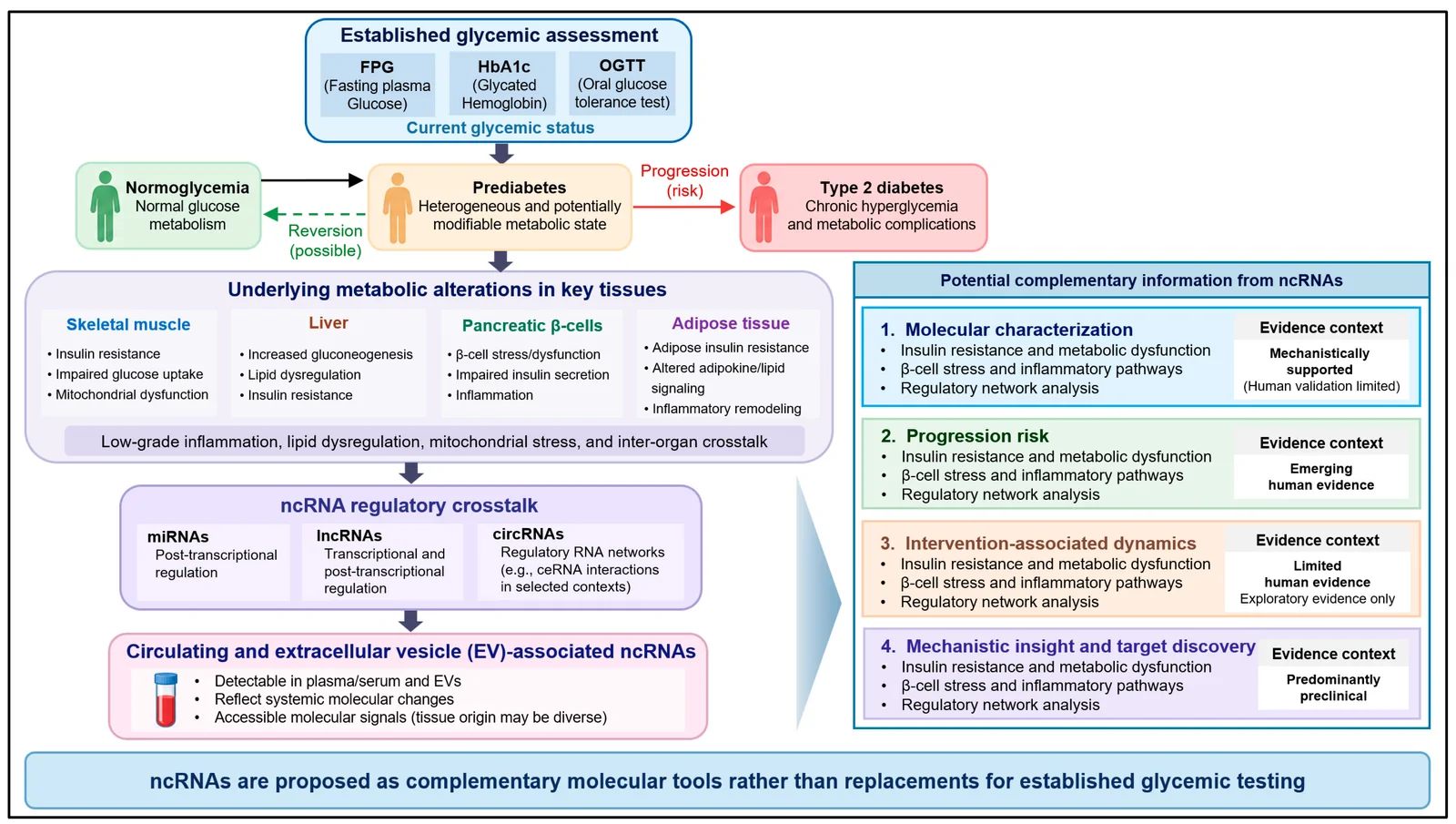
Prediabetes as a modifiable metabolic transition: established glycemic classification and potential complementary roles of non-coding RNAs. Prediabetes represents a heterogeneous and potentially reversible metabolic state between normoglycemia and type 2 diabetes mellitus (T2DM). Established glycemic measurements, including fasting plasma glucose (FPG), glycated hemoglobin (HbA1c), and the oral glucose tolerance test (OGTT), provide the clinical basis for identifying and monitoring prediabetes. However, individuals with similar glycemic profiles may differ in the relative contributions of skeletal muscle insulin resistance, hepatic metabolic dysfunction, adipose tissue insulin resistance and inflammatory remodeling, pancreatic β-cell stress, lipid dysregulation, mitochondrial stress, and inter-organ crosstalk. Non-coding RNAs (ncRNAs), including microRNAs (miRNAs), long non-coding RNAs (lncRNAs), and circular RNAs (circRNAs), participate in regulatory pathways associated with these processes and may also be detected in the circulation, including within extracellular vesicles (EVs). Rather than replacing established glycemic tests, ncRNA profiling may potentially provide complementary information regarding molecular characterization of metabolic dysfunction, future progression risk, intervention-associated molecular responses, and candidate regulatory mechanisms or therapeutic targets. Evidence supporting these applications varies substantially, ranging from direct human prediabetes studies to mechanistic findings derived from related metabolic conditions and experimental models. Accordingly, the proposed roles of ncRNAs remain investigational and require prospective and independent validation.

Fasting plasma glucose (FPG), the oral glucose tolerance test (OGTT), and glycated hemoglobin (HbA1c) provide established, accessible, and clinically validated approaches for identifying prediabetes and monitoring glycemic status [1]. Although analytical, biological, and population-related factors can influence their classification performance [9,10,11], the rationale for developing molecular biomarkers should not primarily be to replace these established tests. Rather, an important unresolved question is whether molecular profiling can provide complementary information that glycemic measurements were not designed to capture directly. Individuals with similar glucose or HbA1c values may differ in the relative contributions of peripheral insulin resistance, hepatic metabolic dysfunction, pancreatic β-cell stress, inflammation, lipid dysregulation, and mitochondrial stress to their metabolic phenotype. Likewise, glycemic classification alone does not directly identify the regulatory pathways underlying these differences or fully characterize molecular changes occurring in response to preventive interventions. Molecular biomarkers may therefore be most informative if they help address questions beyond glycemic classification, including biological heterogeneity, future progression to T2DM, and intervention-associated molecular responses. Figure 1 summarizes this conceptual framework, positioning established glycemic testing as the basis for identifying prediabetes while illustrating the complementary molecular information that ncRNA profiling may potentially provide.

Non-coding RNAs (ncRNAs) are particularly relevant to these questions because they can function both as regulators of metabolic pathways and as measurable molecular signals. MicroRNAs (miRNAs) regulate gene expression post-transcriptionally, whereas long non-coding RNAs (lncRNAs) and circular RNAs (circRNAs) participate in transcriptional, post-transcriptional, and RNA–RNA regulatory processes [12,13,14,15]. These ncRNA classes can also interact through competing endogenous RNA (ceRNA) and related regulatory mechanisms in which lncRNAs or circRNAs modulate miRNA availability and downstream gene expression [15,16]. Experimental studies have linked such regulatory networks to insulin signaling, glucose and lipid metabolism, pancreatic β-cell function, inflammatory responses, and cellular stress. In addition, ncRNAs can be detected in the circulation, either in extracellular vesicles (EVs) or in association with RNA-binding proteins and other extracellular carriers [17]. These properties have led to interest in ncRNAs not only as candidate circulating biomarkers but also as molecular indicators of regulatory processes involved in metabolic deterioration.

However, the evidence base in prediabetes requires careful interpretation. Direct studies in human prediabetes remain substantially less abundant than studies conducted in established T2DM, obesity, insulin resistance, or experimental metabolic models. Moreover, an association between an ncRNA and a metabolic phenotype does not establish a causal regulatory mechanism, and predicted miRNA-binding relationships do not by themselves establish biologically meaningful ceRNA activity. Accordingly, tissue-resolved ncRNA relationships identified in skeletal muscle, liver, pancreatic β-cells, and adipose tissue should not be interpreted as a validated ‘prediabetes-specific interactome’. Instead, they can be integrated into a hypothesis-generating tissue-resolved framework describing regulatory pathways potentially relevant to the prediabetic transition. Such integration is particularly useful when miRNAs, lncRNAs, and circRNAs are considered as interacting regulatory classes rather than as independent lists of candidate biomarkers.

The potential translational value of circulating ncRNAs likewise depends on the clinical question being addressed. Cross-sectional studies have reported miRNAs and circRNAs that discriminate individuals with prediabetes from normoglycemic controls [18,19,20,21,22,23], but diagnostic discrimination alone does not establish that ncRNA testing provides clinically meaningful information beyond FPG, HbA1c, or OGTT. More compelling for future investigation is the possibility that ncRNA profiles may provide prognostic, predictive, or pharmacodynamic information. Prospective studies have identified circulating miRNA signatures associated with subsequent progression to T2DM [24]. Importantly, secondary analyses of the randomized DPP provide a particularly informative translational setting. A baseline 5-miRNA score was associated with incident T2DM and showed an exploratory interaction with randomized intervention assignment, suggesting potential prognostic and treatment-predictive information [25]. In a separate DPP analysis, two years of metformin exposure was associated with changes in multiple circulating miRNAs compared with placebo, providing candidate pharmacodynamic evidence [26]. Although neither finding is sufficient for clinical implementation, together they illustrate how ncRNAs could eventually be evaluated across distinct functions—from identifying progression risk before intervention to characterizing differential intervention benefit and treatment-associated molecular change.

Accordingly, this review evaluates ncRNAs in prediabetes from the perspective of what molecular information they may add beyond glycemic classification, rather than primarily as replacements for established diagnostic tests. We first integrate miRNA-, lncRNA-, and circRNA-mediated pathways across skeletal muscle, liver, pancreatic β-cells, and adipose tissue to construct a tissue-resolved, hypothesis-generating framework of ncRNA crosstalk relevant to early metabolic dysfunction. We then critically evaluate circulating and EV-associated ncRNAs according to distinct biomarker functions, separating cross-sectional diagnostic discrimination from longitudinal prognostic evidence, exploratory treatment-predictive evidence, and pharmacodynamic changes after intervention. Particular attention is given to DPP-derived studies because they connect ncRNA profiles with both diabetes progression and randomized preventive interventions. Finally, we examine intervention-associated ncRNA dynamics, preclinical therapeutic implications, and the analytical and biological barriers that must be addressed before ncRNA profiling can provide clinically meaningful information in prediabetes.

## 2. ncRNA Regulatory Architecture and Crosstalk

Non-coding RNAs (ncRNAs) comprise diverse regulatory molecules that influence gene expression at transcriptional and post-transcriptional levels. Among the ncRNA classes most extensively implicated in metabolic regulation are microRNAs (miRNAs), long non-coding RNAs (lncRNAs), and circular RNAs (circRNAs) [12,13,14,15]. These molecules differ in their biogenesis and modes of action but can converge on common pathways involved in insulin signaling, glucose and lipid metabolism, pancreatic β-cell function, inflammation, and cellular stress. Their potential relevance to prediabetes therefore lies not only in changes in individual ncRNA abundance but also in the regulatory relationships formed among different ncRNA classes.

miRNAs are short regulatory RNAs, typically 19–22 nucleotides in length, that bind partially complementary sequences in target transcripts and promote mRNA degradation or translational repression [12]. Because individual miRNAs can regulate multiple targets within the same signaling pathway, altered miRNA expression can influence coordinated metabolic responses rather than isolated genes. In contrast, lncRNAs are transcripts longer than 200 nucleotides that regulate gene expression through diverse mechanisms, including chromatin remodeling, transcriptional control, interactions with RNA-binding proteins, and modulation of other RNA species [13,15]. CircRNAs are covalently closed transcripts generated by back-splicing and are relatively resistant to exonuclease-mediated degradation [14]. Their stability, together with tissue- and context-dependent expression, has prompted interest in both their regulatory functions and their potential use as circulating biomarkers. An important point of convergence among these ncRNA classes is RNA–RNA crosstalk. LncRNAs and circRNAs can contain microRNA response elements and, in selected molecular contexts, modulate the availability of miRNAs to their target mRNAs, forming competing endogenous RNA (ceRNA) relationships [15,16]. However, the presence of predicted miRNA-binding sites or correlated expression patterns is insufficient to establish a functional ceRNA mechanism. Biologically meaningful competition depends on factors such as transcript abundance, binding affinity, subcellular localization, and the relative stoichiometry of the participating RNAs [27,28,29]. In particular, a putative ceRNA must be present at sufficient abundance relative to the shared miRNA and competing target pool for changes in its expression to measurably alter miRNA availability. Experimental validation should therefore extend beyond predicted binding sites or expression correlations and demonstrate miRNA-dependent and miRNA-response-element-specific regulation at physiologically relevant transcript levels, ideally supported by quantitative abundance measurements and loss-of-function, gain-of-function, and rescue experiments [27,28,29]. Accordingly, ceRNA interactions should be interpreted as experimentally testable, context-dependent regulatory relationships rather than inferred from sequence complementarity alone.

Beyond miRNAs, lncRNAs, and circRNAs, several emerging classes of small ncRNAs are increasingly implicated in metabolic regulation, including PIWI-interacting RNAs (piRNAs), small nucleolar RNAs (snoRNAs), and tRNA-derived fragments (tRFs) [30]. piRNAs are small PIWI-associated RNAs best characterized for transposon silencing and genome protection, although emerging studies suggest broader roles in gene regulation and have identified circulating piRNAs in diabetes-related metabolic phenotypes [30,31]. snoRNAs canonically guide post-transcriptional modification of ribosomal and other RNAs but can also exert noncanonical stress-responsive functions; for example, *Rpl13a*-associated snoRNAs modulate oxidative stress, glucose-stimulated insulin secretion, and systemic glucose tolerance in experimental models [30,32]. tRFs are generated through regulated cleavage of mature or precursor tRNAs and can influence translation, RNA stability, RNA-binding proteins, and stress responses [30]. Of particular relevance to early dysglycemia, recent human islet profiling identified increased 5′tRF^Glu(CTC) in individuals with impaired glucose tolerance, with higher levels associated with reduced insulin secretory function; complementary experimental studies implicated this tRF in β-cell stress and islet macrophage activation [33]. Nevertheless, compared with miRNAs, lncRNAs, and circRNAs, evidence for piRNAs, snoRNAs, and tRFs in human prediabetes remains limited, and their biomarker and mechanistic roles require further prospective validation.

Post-transcriptional RNA modification and editing provide an additional regulatory layer that can influence ncRNA abundance and function. N6-methyladenosine (m6A), one of the most prevalent internal RNA modifications, is dynamically controlled by methyltransferase “writers,” demethylase “erasers,” and m6A-binding “readers” and can regulate RNA processing, stability, localization, and translation [34,35]. Importantly, m6A also occurs on ncRNAs, including lncRNAs, circRNAs, and miRNA precursors, thereby potentially modifying their stability, maturation, and regulatory interactions [35]. Dysregulation of m6A signaling has been linked to β-cell dysfunction, insulin resistance, adipose tissue remodeling, and altered glucose and lipid metabolism in T2DM and related metabolic models [34,36]. A second regulatory mechanism is adenosine-to-inosine (A-to-I) RNA editing, mediated primarily by ADAR enzymes, which can alter RNA secondary structure, splicing, miRNA biogenesis, and miRNA–target recognition [37]. In pancreatic islets and β-cells, ADAR2 expression and A-to-I editing are metabolically responsive to glucose exposure and high-fat-diet-induced insulin resistance, suggesting that RNA editing may participate in nutrient-responsive regulation of β-cell function [37]. However, direct evidence demonstrating specific m6A-modified ncRNAs or A-to-I editing signatures in human prediabetes remains limited. These epitranscriptomic mechanisms should therefore be regarded as emerging regulatory layers relevant to early metabolic dysfunction rather than as validated prediabetes-specific pathways or biomarkers.

This regulatory architecture provides the conceptual basis for the tissue-resolved ncRNA networks examined in subsequent sections. In the context of prediabetes, the key question is not whether the major ncRNA classes emphasized in this review form a single validated disease-specific interactome, but whether selected regulatory relationships identified in skeletal muscle, liver, pancreatic β-cells, adipose tissue, and related metabolic settings can be integrated into a biologically plausible framework for early metabolic dysfunction. The following sections therefore distinguish experimentally supported mechanisms from hypothesis-generating cross-tissue integration and, where possible, separate direct human prediabetes evidence from findings derived from established T2DM or experimental models.

## 3. miRNA Pathways Relevant to Early Metabolic Dysfunction in Prediabetes

miRNAs are among the best-characterized ncRNA regulators of metabolic homeostasis and have been implicated in insulin signaling, pancreatic β-cell adaptation, inflammatory responses, and mitochondrial stress [38,39,40]. These processes are central to the metabolic deterioration that accompanies progression from normoglycemia toward prediabetes and T2DM. However, much of the mechanistic evidence derives from established diabetes, insulin-resistant states, or experimental models rather than from longitudinal studies of human prediabetes. Accordingly, the pathways discussed in this section should be interpreted as biologically relevant mechanisms that may contribute to the prediabetic transition rather than as uniformly validated prediabetes-specific pathways.

**Figure 2:**
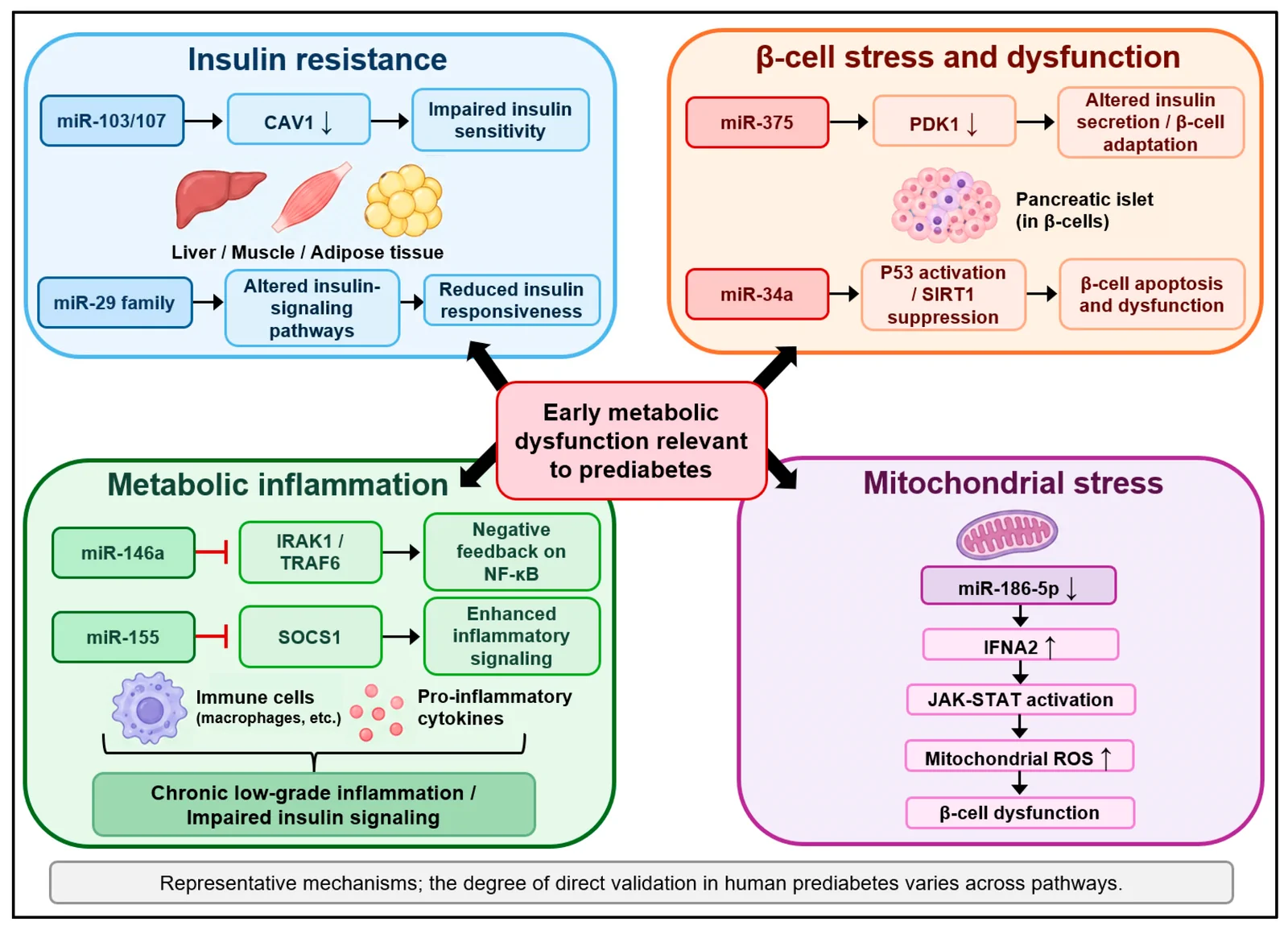
Representative microRNA-mediated pathways relevant to early metabolic dysfunction in prediabetes. Selected microRNAs (miRNAs) are organized according to four interconnected processes implicated in metabolic deterioration relevant to the prediabetic transition: insulin resistance, pancreatic β-cell stress and dysfunction, metabolic inflammation, and mitochondrial stress. In the insulin-resistance branch, the miR-29 family modulates components of insulin-signaling and glucose–lipid metabolic pathways, whereas miR-103/107 suppress caveolin-1 (CAV1), thereby impairing insulin sensitivity. In pancreatic β-cells, miR-375 regulates 3-phosphoinositide-dependent protein kinase-1 (PDK1) and β-cell function, whereas miR-34a is associated with lipotoxic β-cell injury through p53- and sirtuin 1 (SIRT1)-related pathways. In inflammatory signaling, miR-146a provides negative-feedback regulation through interleukin-1 receptor-associated kinase 1 (IRAK1) and TNF receptor-associated factor 6 (TRAF6), whereas miR-155 promotes proinflammatory signaling in part through suppression of suppressor of cytokine signaling 1 (SOCS1). Reduced miR-186-5p under glucolipotoxic conditions has been associated with de-repression of interferon alpha 2 (*IFNA2*), activation of Janus kinase–signal transducer and activator of transcription (JAK–STAT) signaling, and increased mitochondrial reactive oxygen species (ROS) in β-cells. Together, these examples illustrate how dysregulated miRNAs may converge on complementary metabolic pathways involved in early insulin resistance, β-cell dysfunction, inflammation, and cellular stress. The depicted relationships represent mechanistically supported pathways derived from human studies and relevant experimental models; they should not be interpreted as uniformly validated, prediabetes-specific mechanisms. Arrowheads indicate positive or downstream regulatory relationships, whereas T-bars indicate inhibitory effects.

Representative miRNA-mediated mechanisms are summarized in Figure 2, which organizes selected miRNAs according to four interconnected processes relevant to early metabolic dysfunction: insulin resistance, β-cell stress and dysfunction, metabolic inflammation, and mitochondrial stress. This framework is intended to illustrate how distinct miRNAs may converge on complementary metabolic pathways rather than to imply that all depicted relationships have been directly demonstrated in human prediabetes.

### 3.1. Insulin-Resistance-Associated miRNAs

Insulin resistance is a central feature of prediabetes and reflects impaired insulin action across key metabolic tissues, including skeletal muscle, liver, and adipose tissue [2,3,41]. Several miRNAs have been shown to regulate components of the insulin-signaling pathway and may therefore contribute to the development or maintenance of insulin-resistant states. The insulin-resistance branch of this regulatory framework is summarized in Figure 2.

Among the most extensively studied examples are miR-103 and miR-107. In experimental models, increased miR-103/107 activity has been associated with impaired insulin sensitivity through repression of caveolin-1 (CAV1), a membrane-associated scaffolding protein involved in insulin receptor organization and signaling [39]. Experimental inhibition of miR-103/107 increased CAV1 expression and improved insulin sensitivity, supporting a functional role for this miRNA family in metabolic regulation [39]. Although these findings provide strong mechanistic evidence, direct demonstration of the miR-103/107–CAV1 axis as a driver of human prediabetes remains limited. The miR-29 family represents another regulatory node linked to insulin resistance. Altered miR-29 expression has been reported in insulin-resistant and diabetic states, and experimental studies indicate that miR-29 family members can influence components of insulin signaling and glucose and lipid metabolism in skeletal muscle [42,43]. These findings support the possibility that dysregulated miR-29 signaling contributes to reduced insulin responsiveness during metabolic stress. However, the direction and magnitude of miR-29 changes may vary according to tissue, metabolic context, and disease stage, and evidence specifically establishing its role in human prediabetes remains incomplete. Members of the let-7 family have also been implicated in glucose homeostasis and insulin sensitivity. Experimental manipulation of let-7 signaling alters the expression of genes involved in insulin receptor signaling and glucose metabolism [44]. In addition, increased circulating let-7i-5p has been reported in obese women with impaired fasting glucose, providing direct human evidence that selected let-7 family members may be associated with prediabetic metabolic status [19]. Nevertheless, circulating associations should not be assumed to directly reflect tissue-level regulatory activity.

Collectively, these findings indicate that miRNAs such as miR-103/107, miR-29 family members, and let-7 can modulate insulin-signaling pathways relevant to early metabolic dysfunction. Their principal value in the context of this review is therefore mechanistic: they illustrate how post-transcriptional regulation may contribute to heterogeneity in insulin resistance during the prediabetic transition. Whether these pathways can provide clinically useful information beyond conventional measures of glycemia and insulin resistance remains to be established.

### 3.2. β-Cell-Stress- and Dysfunction-Associated miRNAs

Progressive pancreatic β-cell dysfunction is a key determinant of the transition from compensated insulin resistance to overt dysglycemia [2,3]. During early metabolic stress, β-cells initially increase insulin secretion to compensate for reduced peripheral insulin sensitivity; however, sustained glucolipotoxic, inflammatory, and oxidative stress can impair β-cell adaptation and ultimately compromise insulin secretion [20,45,46]. Several miRNAs participate in pathways relevant to β-cell survival, secretory function, and stress responses [20,38,45,46,47,48,49,50], as summarized in the β-cell and mitochondrial-stress branches of Figure 2.

Among the best-characterized β-cell miRNAs, miR-375 is highly expressed in pancreatic islets and plays an important role in insulin secretion and maintenance of β-cell mass [38,50]. However, high islet abundance should not be equated with exclusive β-cell specificity in the circulation. Experimental studies indicate that β-cells contribute only a fraction of basal circulating miR-375, although acute β-cell injury can produce measurable increases in plasma miR-375 [51,52,53]. One experimentally validated target is 3-phosphoinositide-dependent protein kinase-1 (PDK1), a component of intracellular signaling pathways involved in β-cell function [49]. Experimental manipulation of miR-375 alters insulin secretion and β-cell mass, supporting a direct regulatory role in pancreatic endocrine homeostasis [38,49,50]. However, most mechanistic evidence for this pathway derives from experimental models, and its specific contribution to human prediabetes remains to be defined. miR-34a represents another stress-responsive miRNA implicated in β-cell injury. Under lipotoxic conditions, miR-34a expression increases and has been associated with impaired insulin secretion and β-cell apoptosis [45]. This effect involves activation of p53-related pro-apoptotic pathways and suppression of sirtuin 1 (SIRT1), a regulator of cellular survival and metabolic adaptation [45,48]. These findings provide a mechanistic link between lipid-induced cellular stress and miRNA-mediated β-cell dysfunction, but they should be interpreted primarily as experimental evidence relevant to the prediabetic transition rather than as a validated human prediabetes-specific pathway. miR-186-5p provides a more direct link between human prediabetes-associated circulating signals and β-cell stress mechanisms. Plasma extracellular vesicle (EV)-associated miR-186-5p was reduced in individuals with prediabetes, and complementary experimental studies showed that reduced miR-186-5p under glucolipotoxic conditions was associated with de-repression of IFN-α (*IFNA2*), activation of JAK–STAT signaling, increased mitochondrial reactive oxygen species, and impaired β-cell function [20]. Restoration of miR-186-5p partially attenuated these stress responses in experimental β-cell models [20]. This combination of human association and mechanistic evidence makes miR-186-5p a useful example of how circulating ncRNA signals may be linked to biologically plausible pathways relevant to early β-cell dysfunction. Nevertheless, the circulating signal does not by itself establish pancreatic β-cells as its exclusive tissue of origin.

Collectively, miR-375, miR-34a, and miR-186-5p illustrate distinct mechanisms through which miRNAs may influence β-cell adaptation, survival, and mitochondrial stress during metabolic deterioration. Their principal relevance to prediabetes is currently mechanistic and hypothesis-generating, with direct longitudinal evidence linking these pathways to progression from prediabetes to T2DM still limited.

### 3.3. Inflammation-Associated miRNAs

Chronic low-grade inflammation contributes to the development of insulin resistance and β-cell stress and is increasingly recognized as part of the metabolic environment preceding overt T2DM [54,55]. miRNAs can modulate this inflammatory state by regulating signaling pathways in immune cells and metabolically active tissues. Among the most extensively studied examples are miR-146a and miR-155, which exert partially opposing effects on inflammatory signaling and are summarized in Figure 2.

miR-146a functions as a negative-feedback regulator of inflammatory signaling by targeting interleukin-1 receptor-associated kinase 1 (IRAK1) and TNF receptor-associated factor 6 (TRAF6), thereby limiting activation of nuclear factor-κB (NF-κB) pathways [47]. Reduced miR-146a expression has been associated with impaired inflammatory feedback, increased circulating inflammatory mediators, and insulin resistance in individuals with metabolic disease [47]. In pancreatic β-cells, proinflammatory cytokines can also modify miR-146a expression, linking inflammatory exposure to β-cell stress responses [46]. These observations support a model in which disruption of miR-146a-mediated negative feedback may contribute to sustained inflammatory signaling during metabolic stress. In contrast, miR-155 is generally associated with amplification of proinflammatory signaling. One reported mechanism involves suppression of suppressor of cytokine signaling 1 (SOCS1), which can enhance downstream STAT signaling and inflammatory mediator production [56]. Increased miR-155 activity has therefore been linked to inflammatory states that may further impair insulin signaling and contribute to metabolic dysfunction [56,57]. The functional balance between miR-146a-mediated negative feedback and miR-155-associated inflammatory amplification provides a useful framework for understanding how miRNA dysregulation may influence the inflammatory component of insulin resistance. Evidence directly establishing these inflammatory miRNA pathways in longitudinal human prediabetes remains limited. Most mechanistic support derives from T2DM, inflammatory disease settings, or experimental systems. Accordingly, miR-146a and miR-155 should be viewed as representative regulators of inflammatory pathways relevant to early metabolic dysfunction rather than as established prediabetes-specific biomarkers or causal drivers. Their potential clinical significance will depend on whether future studies demonstrate reproducible associations with progression risk or intervention-associated molecular changes beyond conventional inflammatory and metabolic measures.

### 3.4. Circulating miRNAs as Extracellular Correlates of Metabolic Dysfunction

miRNAs involved in intracellular metabolic regulation can also be detected in the circulation, either within EVs or in association with RNA-binding proteins and other extracellular carriers [17,58]. Their extracellular stability and accessibility have generated substantial interest in circulating miRNAs as candidate biomarkers of metabolic dysfunction [59]. Importantly, tissue enrichment and circulating stability represent distinct properties of a candidate miRNA biomarker. A miRNA that is highly abundant in a particular tissue may have biological plausibility as a tissue-associated signal, whereas its detectability in blood depends on its release into the extracellular compartment and subsequent protection from degradation. Circulating miRNAs can remain relatively stable because they are encapsulated within EVs or associated with RNA-binding proteins such as Argonaute proteins and with lipoprotein particles, which can protect them from extracellular RNases [17,58]. However, such extracellular stabilization does not preserve or establish tissue specificity. Accordingly, circulating abundance should not be assumed to directly reflect intracellular expression in a specific tissue, because extracellular miRNA levels are influenced by multiple processes, including active secretion, EV packaging, cellular injury, and association with extracellular protein carriers [17,52,58,60].

Several miRNAs implicated in insulin resistance have also been detected in the circulation. Members of the miR-29 and miR-103/107 families have been investigated in both tissue and circulating contexts, although concordance between intracellular and circulating levels is not consistent across studies [39,42,59]. Similarly, selected let-7 family members have been associated with insulin-signaling pathways in experimental models, while circulating let-7i-5p was increased in obese women with impaired fasting glucose [19,44]. These observations support biological relevance but do not establish that circulating levels provide a direct readout of skeletal muscle, hepatic, or adipose-tissue activity. A similar distinction applies to β-cell-associated miRNAs. Although miR-375 is highly enriched in pancreatic islets, this tissue enrichment does not imply that circulating miR-375 is exclusively β-cell-derived. Experimental studies indicate that acute β-cell injury can increase circulating miR-375, whereas β-cells account for only a limited proportion of basal circulating miR-375, underscoring the distinction between tissue enrichment and circulating tissue specificity [51,52,53]. However, plasma EV-associated miR-186-5p has shown direct human association with prediabetes and complementary mechanistic links to IFN-α/JAK–STAT signaling and mitochondrial oxidative stress in β-cells [20]. The latter illustrates how a circulating ncRNA signal can be connected to a plausible intracellular pathway while still requiring caution regarding tissue attribution. EV-associated miRNAs are of particular interest because EV cargo can be selectively packaged and released under conditions of cellular stress [17]. This regulated process may enrich circulating RNA profiles for biologically informative signals. Nevertheless, the detection of a miRNA within circulating EVs does not by itself identify the tissue or cell type from which the EV originated. Therefore, EV-associated miRNAs should be regarded as potentially tissue-informative rather than tissue-specific unless supported by additional experimental or molecular tracing evidence.

Single-cell and spatial transcriptomic approaches may help address this tissue-of-origin uncertainty by providing cell-type-resolved and anatomically resolved reference maps for candidate ncRNAs and their associated regulatory programs. Single-cell transcriptomics can identify specific cellular populations and metabolic cell states in tissues such as skeletal muscle, liver, pancreatic islets, and adipose tissue, whereas spatial transcriptomics can retain information on tissue architecture and local cellular neighborhoods [61]. However, conventional single-cell RNA-sequencing and most current spatial transcriptomic platforms are optimized primarily for longer or polyadenylated transcripts and are not designed for comprehensive detection of mature miRNAs and other small RNAs. Emerging single-cell small-RNA sequencing methods can directly profile miRNAs and other small ncRNAs at cellular resolution [62,63], while newer spatial approaches are beginning to extend transcriptomic mapping toward miRNA and other ncRNA species [64,65]. These technologies could therefore help prioritize the most plausible cellular sources of circulating ncRNA candidates, but tissue-of-origin assignment would still require validation using paired tissue and biofluid measurements, EV characterization, or experimental tracing approaches.

Taken together, circulating miRNAs may provide accessible extracellular correlates of metabolic processes involving insulin resistance, β-cell stress, inflammation, and mitochondrial dysfunction. Their potential value in prediabetes may therefore extend beyond cross-sectional discrimination toward molecular characterization, progression risk assessment, and intervention-associated monitoring. However, these applications require longitudinal validation and demonstration of added information beyond established glycemic and clinical risk measures. The biomarker evidence supporting these potential roles is evaluated separately in the subsequent sections.

## 4. Tissue-Resolved ncRNA Crosstalk Relevant to the Prediabetic Transition

The metabolic abnormalities that characterize prediabetes arise from coordinated dysfunction across multiple tissues, including skeletal muscle, liver, pancreatic β-cells, and adipose tissue [2,41]. ncRNAs may contribute to this inter-tissue heterogeneity through tissue-dependent regulatory mechanisms involving miRNAs, lncRNAs, and circRNAs. Rather than acting as isolated molecular species, these ncRNA classes can participate in interconnected regulatory relationships that influence insulin signaling, lipid metabolism, β-cell function, inflammatory responses, and cellular stress [15,16]. Integrating these relationships across metabolically relevant tissues can therefore provide a useful framework for examining how distinct molecular processes may contribute to the transition from compensated metabolic stress to overt dysglycemia. However, the available evidence does not support the existence of a single, experimentally validated “prediabetes-specific ncRNA interactome.” Much of the mechanistic evidence has been generated in established T2DM, obesity, insulin-resistant states, cultured cells, or animal models. Direct evidence demonstrating coordinated ncRNA network changes in human prediabetes is comparatively limited. Accordingly, this section does not attempt to define a validated disease-specific interactome. Instead, it integrates representative tissue-resolved regulatory relationships into a hypothesis-generating framework relevant to the prediabetic transition, while distinguishing experimentally supported mechanisms from context-dependent or inferential connections.

Throughout this section, evidence is interpreted according to its experimental and clinical context. Direct human prediabetes evidence refers to ncRNA associations demonstrated in cohorts meeting established criteria for prediabetes, whereas supportive human evidence includes findings from individuals with established T2DM, obesity, or insulin resistance. Mechanistic evidence derived from cultured cells or animal models is considered separately and is used to identify biologically plausible regulatory pathways rather than to establish prediabetes-specific mechanisms. Accordingly, the tissue-resolved networks described below should be regarded as a hypothesis-generating framework integrating evidence of different levels of directness, rather than as a regulatory network that has been validated specifically in human prediabetes.

**Figure 3:**
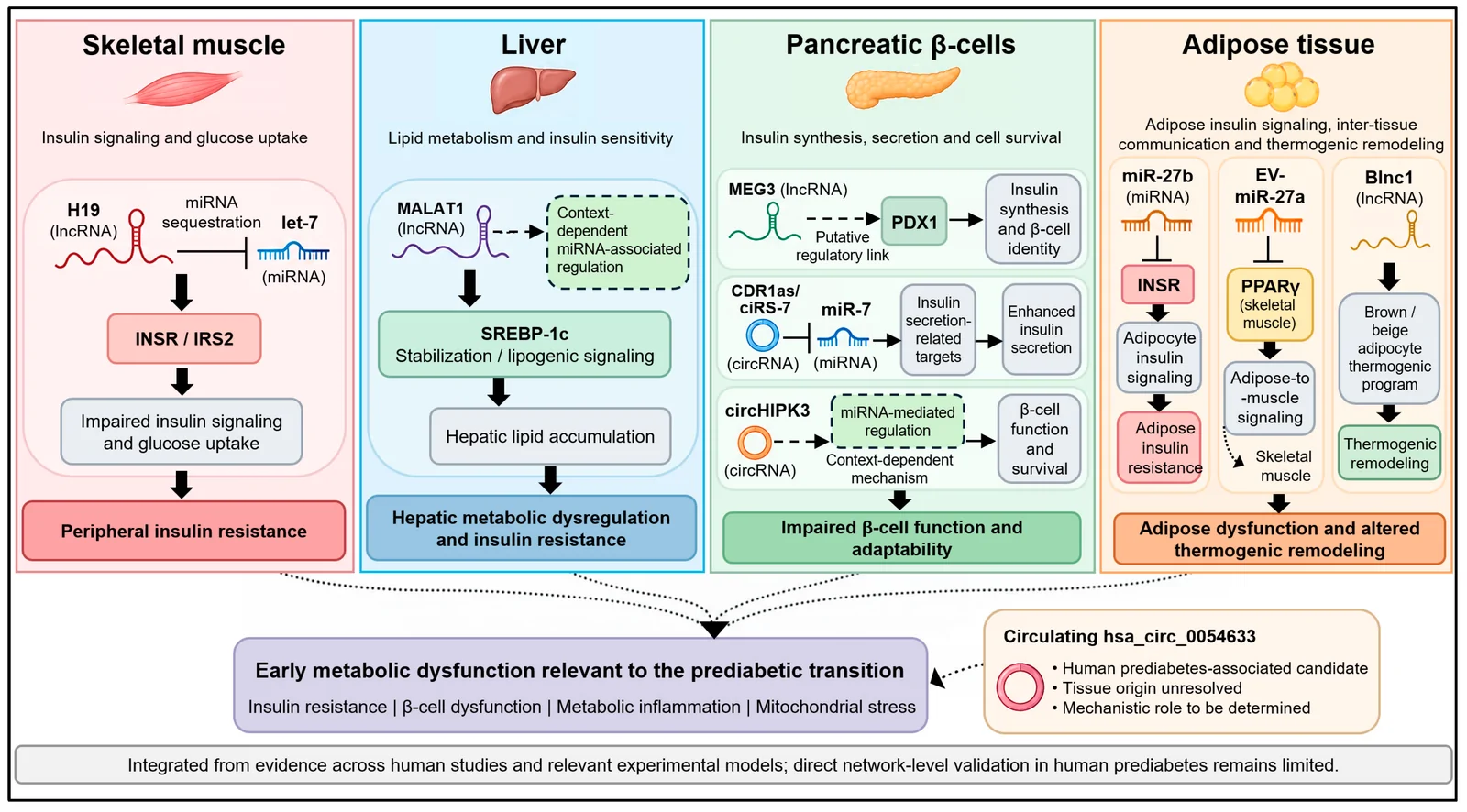
Hypothesis-generating tissue-resolved ncRNA crosstalk framework relevant to the prediabetic transition. Representative ncRNA regulatory pathways are organized across four metabolically relevant compartments: skeletal muscle, liver, pancreatic β-cells, and adipose tissue. In skeletal muscle, the *H19*/let-7 axis represents lncRNA–miRNA regulation of insulin signaling. In the liver, *MALAT1*-associated regulation illustrates ncRNA involvement in lipogenic signaling and hepatic insulin resistance. In pancreatic β-cells, *MEG3*-associated PDX1 regulation and circRNA pathways involving CDR1as/ciRS-7 and circHIPK3 represent mechanisms related to β-cell identity, insulin secretion, and survival. In adipose tissue, miR-27b-associated repression of *INSR* represents adipocyte-autonomous regulation of insulin signaling, adipose-derived EV-miR-27a-associated repression of PPARγ illustrates adipose-to-muscle inter-tissue communication, and *Blnc1*-dependent regulation represents control of brown/beige adipocyte thermogenic remodeling. Solid arrows indicate experimentally supported relationships, dashed arrows indicate putative or context-dependent regulatory relationships, and dotted arrows indicate hypothesis-generating integrative links. Arrowheads denote positive or downstream relationships, whereas T-bars denote inhibitory or miRNA-sequestering effects. Circulating hsa_circ_0054633 is shown separately because its tissue origin and mechanistic placement remain unresolved. Overall, the figure integrates evidence from human studies and relevant experimental models and should not be interpreted as a validated prediabetes-specific ncRNA network. ncRNA, non-coding RNA.

The framework summarized in Figure 3 focuses on four metabolically relevant compartments: skeletal muscle, liver, pancreatic β-cells, and adipose tissue. In skeletal muscle, the *H19*/let-7 axis illustrates how lncRNA–miRNA crosstalk may influence insulin signaling [66,67]. In the liver, *MALAT1*-associated regulation provides an example of ncRNA involvement in lipogenic and insulin-resistant pathways, although a specific *MALAT1*-centered ceRNA mechanism is not established in human prediabetes [68]. In pancreatic β-cells, *MEG3*-associated regulation of PDX1 and circRNA-mediated pathways involving CDR1as/ciRS-7 and circHIPK3 illustrate potential ncRNA control of β-cell identity, insulin secretion, and survival [69,70,71]. In adipose tissue, miR-27-associated regulation of adipocyte insulin signaling and inter-tissue communication, together with *Blnc1*-dependent control of brown/beige adipocyte thermogenesis and remodeling, illustrates complementary ncRNA mechanisms relevant to metabolic homeostasis [72,73,74]. These representative axes are used to organize mechanistic evidence across tissues rather than to imply that they constitute a single validated network in human prediabetes.

### 4.1. ceRNA Crosstalk: Mechanistic Requirements and Evidence Boundaries

One mechanism through which different ncRNA classes can interact is the competing endogenous RNA (ceRNA) model. In this framework, lncRNAs, circRNAs, and other transcripts containing shared microRNA response elements (MREs) can compete for binding to specific miRNAs, thereby altering the availability of those miRNAs for conventional mRNA targets [16]. In principle, such competition can modify downstream gene expression without changing transcription of the target gene itself.

Importantly, the presence of a predicted miRNA-binding site does not by itself establish biologically meaningful ceRNA activity. Quantitative studies have shown that effective competition depends on several factors, including the relative abundance of the participating RNAs, the number and affinity of MREs, miRNA concentration, and subcellular co-localization [27,28,29]. Consequently, sequence complementarity, shared MREs, or correlated expression patterns should be considered hypothesis-generating evidence rather than proof of ceRNA regulation. Establishing a functional ceRNA relationship requires experimental demonstration that altering one RNA changes the availability or activity of the shared miRNA and produces reciprocal effects on downstream targets or relevant cellular phenotypes. These requirements are especially important when interpreting metabolic ncRNA networks. Numerous lncRNAs and circRNAs have been reported to change under nutrient excess, lipotoxicity, inflammatory stress, or insulin-resistant conditions, and some are associated with pathways relevant to early dysglycemia [18,66,68]. However, such observations do not establish that the reported RNAs operate through ceRNA mechanisms in human prediabetes. Similarly, evidence supporting one component of a regulatory axis in skeletal muscle, liver, or β-cells should not automatically be extrapolated to a coordinated cross-tissue network.

Accordingly, the tissue-resolved framework used in this review applies a graded interpretation of ncRNA crosstalk. Experimentally validated RNA–RNA or RNA–target relationships are distinguished from supportive but context-dependent associations, while cross-tissue integration is treated as hypothesis-generating. This distinction is reflected in Figure 3, in which solid connections denote experimentally supported relationships in relevant metabolic models, dashed connections indicate putative or context-dependent interactions, and dotted connections represent integrative links proposed for the prediabetes framework. Direct longitudinal evidence showing that coordinated ncRNA crosstalk precedes or predicts changes in fasting glucose, HbA1c, or progression to T2DM remains limited and represents a major area for future investigation.

### 4.2. Representative ncRNA Regulatory Axes Across Metabolic Tissues

The tissue-resolved framework described above is supported by several representative ncRNA-centered regulatory axes that influence metabolic pathways relevant to insulin resistance and β-cell dysfunction [66,68,69]. However, the strength and directness of the evidence differ substantially across tissues, and most of these mechanisms have not been directly validated in human prediabetes. Accordingly, the examples discussed below are selected to illustrate how ncRNA-mediated regulation may contribute to distinct components of early metabolic dysfunction rather than to define a unified prediabetes-specific network. In skeletal muscle, the *H19*/let-7 axis provides one of the most mechanistically characterized examples of lncRNA–miRNA crosstalk affecting insulin signaling [66]. In the liver, *MALAT1* is linked to SREBP-1c-dependent lipogenic signaling and hepatic metabolic dysfunction [68], although its contribution through a specific ceRNA mechanism remains context-dependent. In pancreatic β-cells, *MEG3* is associated with PDX1-dependent programs involved in insulin synthesis, secretion, and cell survival [69], but a defined *MEG3*–miRNA–PDX1 ceRNA axis has not been established. In adipose tissue, miR-27-associated regulation of adipocyte insulin signaling and inter-tissue communication, together with *Blnc1*-dependent control of brown/beige adipocyte thermogenesis and remodeling, illustrates complementary ncRNA mechanisms relevant to metabolic homeostasis [72,73,74]. Together, these examples illustrate tissue-dependent modes of ncRNA regulation while also highlighting the current gap between mechanistic evidence from experimental systems and direct network-level validation in human prediabetes.

#### 4.2.1. Skeletal Muscle: *H19*/let-7 Regulation of Insulin Signaling

*H19* is one of the best-characterized lncRNAs implicated in skeletal muscle glucose metabolism and insulin sensitivity. Mechanistically, *H19* interacts with members of the let-7 miRNA family, which regulate multiple components of the insulin-signaling pathway [66,67]. By sequestering let-7 miRNAs, *H19* can reduce let-7-mediated repression of targets involved in insulin action, including the insulin receptor (*INSR*) and insulin receptor substrate 2 (*IRS2*) [66,67]. This relationship provides a well-supported example of lncRNA–miRNA crosstalk with functional consequences for metabolic signaling. Experimental studies further indicate that reduced *H19* expression can impair insulin responsiveness in skeletal muscle. Loss of *H19* increases the availability of let-7 miRNAs and is associated with reduced expression of insulin-signaling components and diminished insulin sensitivity [66]. *H19* has also been linked to AMP-activated protein kinase (AMPK)-dependent regulation of skeletal muscle insulin sensitivity, suggesting that its metabolic effects may extend beyond a single ceRNA interaction [75]. Together, these findings support *H19* as a mechanistically plausible regulator of peripheral insulin sensitivity through both let-7-dependent and additional metabolic pathways. Among the tissue-resolved examples discussed in this review, the *H19*/let-7 axis represents one of the more experimentally substantiated ncRNA regulatory relationships. However, its role has been established predominantly in experimental and insulin-resistant settings rather than through direct longitudinal studies of human prediabetes. The axis should therefore be considered a mechanistic model relevant to the early development of peripheral insulin resistance, rather than a validated prediabetes-specific pathway.

#### 4.2.2. Liver: *MALAT1*-Associated Regulation of Lipid Metabolism

*MALAT1* (metastasis-associated lung adenocarcinoma transcript 1) is a highly conserved lncRNA implicated in hepatic lipid metabolism and metabolic stress [68]. In experimental hepatocyte and metabolic disease models, increased *MALAT1* expression has been linked to stabilization of nuclear sterol regulatory element-binding protein 1c (SREBP-1c), a major transcriptional regulator of de novo lipogenesis [68]. Through this mechanism, *MALAT1* promotes lipogenic gene expression and hepatic lipid accumulation, processes closely associated with hepatic insulin resistance and metabolic dysfunction. Unlike the *H19*/let-7 axis, the metabolic effects of *MALAT1* should not be interpreted primarily as an established ceRNA mechanism. *MALAT1* can regulate gene expression through several modes of action, including interactions with RNA-binding proteins, modulation of RNA stability, transcriptional regulation, and, in selected experimental contexts, miRNA-associated interactions [15,68]. However, a specific *MALAT1*-centered miRNA or ceRNA axis that is reproducibly operative in human prediabetes has not been established. For this reason, Figure 3 depicts miRNA-associated regulation of *MALAT1* as context-dependent rather than as a validated component of a prediabetes-specific ceRNA network. *MALAT1* has also been linked to inflammatory and endothelial responses under hyperglycemic conditions [76], suggesting that its metabolic relevance may extend beyond hepatocyte lipid metabolism. Nevertheless, the strongest mechanistic rationale within the present tissue-resolved framework is its association with SREBP-1c-dependent lipogenic signaling and hepatic lipid accumulation. These findings support *MALAT1* as a candidate regulator of hepatic metabolic dysfunction relevant to the prediabetic transition, while direct validation in human prediabetes remains limited.

#### 4.2.3. Pancreatic β-Cells: *MEG3*-Associated Regulation of PDX1

Maternally expressed gene 3 (*MEG3*) is a lncRNA expressed in pancreatic β-cells and has been implicated in the regulation of β-cell identity, insulin synthesis, and cell survival [69]. Experimental downregulation of *MEG3* in mouse pancreatic β-cells was associated with reduced expression of pancreatic and duodenal homeobox 1 (PDX1), impaired insulin synthesis and secretion, and increased β-cell apoptosis [69]. Because PDX1 is a central transcriptional regulator of β-cell identity and insulin gene expression, these findings support a functional relationship between *MEG3* expression and PDX1-dependent β-cell programs. The molecular mechanism linking *MEG3* to PDX1, however, remains incompletely defined. Although lncRNAs can modulate gene expression through miRNA-dependent and ceRNA-like mechanisms [15,16], the available evidence does not establish a specific *MEG3*–miRNA–PDX1 ceRNA axis in pancreatic β-cells. Accordingly, the *MEG3*–PDX1 relationship is represented in Figure 3 as a putative regulatory link rather than as a validated RNA–RNA interaction. From the perspective of prediabetes, *MEG3* is therefore most appropriately viewed as a candidate regulator of β-cell functional integrity rather than as an established biomarker or disease-specific network component. Direct studies are needed to determine whether *MEG3* expression changes during human prediabetes, whether such changes precede deterioration in β-cell function, and whether they contribute causally to progression toward T2DM.

Beyond *H19*, *MALAT1*, and *MEG3*, additional lncRNAs have been implicated in insulin resistance, lipid dysregulation, β-cell dysfunction, inflammation, and diabetic complications. However, direct evidence linking these candidates to human prediabetes remains sparse, and many reported associations derive from established T2DM or experimental models. At present, these additional lncRNAs are therefore better regarded as exploratory candidates for future tissue-specific and longitudinal validation rather than as established components of a prediabetes regulatory network. Collectively, the *H19*-, *MALAT1*-, and *MEG3*-centered examples illustrate that lncRNA regulation is highly tissue- and context-dependent and can operate through mechanisms extending beyond classical ceRNA competition. Their value in the present framework lies primarily in identifying biologically plausible regulatory pathways that may contribute to heterogeneity in early metabolic dysfunction and that warrant direct investigation in human prediabetes.

#### 4.2.4. Adipose Tissue: ncRNA Regulation of Insulin Sensitivity and Thermogenic Remodeling

Adipose tissue is a heterogeneous metabolic and endocrine organ that contributes to systemic insulin sensitivity, lipid handling, inflammatory signaling, and inter-organ metabolic communication [77,78]. White adipose tissue (WAT) primarily serves as an energy-storage and endocrine compartment, whereas brown adipose tissue (BAT) and inducible beige adipocytes contribute to energy expenditure through mitochondrial thermogenesis [77,79]. Importantly, WAT is itself metabolically heterogeneous across anatomical depots. Visceral WAT is more strongly associated with increased lipolytic activity, inflammatory signaling, and systemic insulin resistance, whereas subcutaneous WAT generally provides greater capacity for metabolically safer lipid storage and can support beige adipocyte recruitment, although these depot-specific effects remain context dependent [78]. Dysfunctional adipose tissue expansion is accompanied by adipocyte hypertrophy, immune-cell recruitment, altered adipokine and lipid release, and impaired insulin action, while reduced brown/beige adipocyte activity may further compromise systemic metabolic homeostasis [77,78]. ncRNAs participate in these processes and in adipose-derived intercellular communication, providing a mechanistic link between adipose tissue dysfunction and the metabolic abnormalities relevant to the prediabetic transition [80].

The miR-27 family provides a representative example of adipose-associated ncRNA regulation. In experimental models of insulin resistance, miR-27b is increased in insulin-resistant adipocytes and directly targets the insulin receptor (*INSR*); its overexpression impairs insulin signaling and glucose uptake, whereas miR-27b inhibition improves adipose insulin sensitivity and glucose tolerance in high-fat-diet-fed mice [72]. Adipose-derived miRNAs may also mediate inter-organ communication. Yu et al. demonstrated that adipocyte-derived exosomal miR-27a can impair insulin signaling in skeletal muscle through repression of PPARγ, providing a mechanistic example of adipose-to-muscle ncRNA crosstalk [73]. Notably, circulating miR-27a was also elevated in obese individuals with prediabetes or T2DM in that study, although these findings do not establish miR-27a as a prediabetes-specific biomarker or demonstrate that the same mechanism operates longitudinally during progression to T2DM [73]. More broadly, adipose-derived extracellular-vesicle-associated ncRNAs have emerged as potential mediators of communication between adipose tissue and other insulin-responsive organs, although their causal contribution in human prediabetes remains incompletely defined [80].

lncRNAs provide an additional regulatory layer, particularly in thermogenic adipose tissue. Brown fat lncRNA 1 (*Blnc1*) promotes brown/beige adipocyte programs and contributes to thermogenic and homeostatic adipose remodeling. In adipose-specific mouse models, loss of *Blnc1* impaired cold-induced thermogenesis and WAT browning and exacerbated obesity-associated adipose inflammation and fibrosis, insulin resistance, and hepatic steatosis, whereas adipose *Blnc1* overexpression produced reciprocal metabolically favorable effects [74]. These observations illustrate how lncRNA-dependent regulation of brown/beige adipocyte function may influence systemic metabolic homeostasis in addition to the miRNA-mediated regulation of insulin signaling observed in WAT.

Together, these findings position adipose ncRNA networks at the intersection of energy storage, inflammatory signaling, insulin responsiveness, thermogenesis, and inter-organ communication. However, most mechanistic relationships described above have been established in cultured adipocytes, animal models, or obesity and insulin-resistant states rather than in longitudinal human prediabetes cohorts. They should therefore be interpreted as biologically plausible mechanisms relevant to the prediabetic transition rather than as validated adipose ncRNA networks specific to human prediabetes.

### 4.3. circRNA-Mediated Regulation and Circulating Biomarker Relevance

Circular RNAs (circRNAs) represent a distinct class of regulatory ncRNAs characterized by covalently closed structures generated through back-splicing [14]. Their relative resistance to exonuclease-mediated degradation contributes to greater molecular stability than many linear RNA species, supporting both intracellular regulatory functions and potential applications as circulating biomarkers. In metabolic tissues, circRNAs can influence gene expression through interactions with miRNAs and RNA-binding proteins, although functional effects vary according to cellular context and should not be inferred solely from predicted binding relationships.

Among metabolically relevant circRNAs, CDR1as, also known as ciRS-7, provides one of the clearest examples of circRNA–miRNA crosstalk. CDR1as contains multiple binding sites for miR-7 and can modulate the availability of miR-7 to downstream targets [70]. In pancreatic islet cells, this regulatory relationship has been linked to genes involved in insulin transcription and secretion, supporting a functional role for the CDR1as/miR-7 axis in β-cell activity [70]. Within the present framework, this axis is therefore included as a mechanistically supported example of circRNA-mediated regulation relevant to β-cell function, although direct validation in human prediabetes remains limited. CircHIPK3 represents another circRNA implicated in β-cell regulation. Experimental studies have linked circHIPK3 to miRNA-mediated pathways involved in insulin secretion, β-cell function, and cell survival [71]. However, the specific miRNA interactions and downstream consequences appear to vary by experimental context. Accordingly, circHIPK3 is best interpreted as a context-dependent regulatory candidate rather than as a component of an established prediabetes-specific ceRNA network. This distinction is reflected in Figure 3, where circHIPK3-associated regulation is depicted as supportive or context-dependent rather than uniformly validated. In addition to these mechanistically characterized circRNAs, hsa_circ_0054633 provides direct human evidence as a circulating candidate associated with prediabetes. Its expression in peripheral blood differed among normoglycemic individuals, individuals with prediabetes, and patients with T2DM, with preliminary discriminative performance across glycemic states [18]. However, its tissue of origin, intracellular function, and placement within a tissue-specific regulatory network remain unresolved. For this reason, hsa_circ_0054633 is presented separately in Figure 3 as a human prediabetes-associated circulating candidate rather than as a mechanistically resolved tissue node.

Together, CDR1as/ciRS-7, circHIPK3, and hsa_circ_0054633 illustrate two complementary aspects of circRNA biology relevant to prediabetes: mechanistic regulation within metabolic tissues and accessibility as circulating molecular signals. At present, however, these roles remain supported by different levels of evidence and should not be conflated. Tissue-level circRNA mechanisms require direct validation in human prediabetes, whereas circulating circRNA associations require replication, analytical standardization, and demonstration of added value beyond established glycemic and clinical risk measures. The next section therefore considers circulating ncRNAs separately according to their potential biomarker functions.

## 5. What Can Circulating ncRNAs Add Beyond Glycemic Classification?

Circulating ncRNAs have attracted considerable interest as minimally invasive biomarkers because they can be measured in plasma, serum, whole blood, or extracellular vesicle (EV) fractions and may reflect molecular processes associated with metabolic dysfunction [17,59]. However, the clinical relevance of these molecules depends on the specific biomarker question being addressed. In prediabetes, established glycemic tests already provide accessible and validated approaches for identifying dysglycemia [1]. Therefore, the value of circulating ncRNAs should not be judged solely by their ability to distinguish prediabetes from normoglycemia, but by whether they provide complementary information beyond conventional glycemic classification.

For this reason, biomarker terminology in this review is used according to study design and intended clinical function [81]. Diagnostic or discriminative biomarkers distinguish an existing metabolic state; prognostic biomarkers are associated with a future clinical outcome irrespective of treatment; predictive biomarkers identify differential benefit from a specific intervention and therefore require a treatment-by-biomarker interaction; and pharmacodynamic biomarkers reflect molecular changes occurring after an intervention. These categories should not be used interchangeably. In particular, cross-sectional discrimination does not establish temporal precedence, future disease risk, or treatment responsiveness.

**Figure 4:**
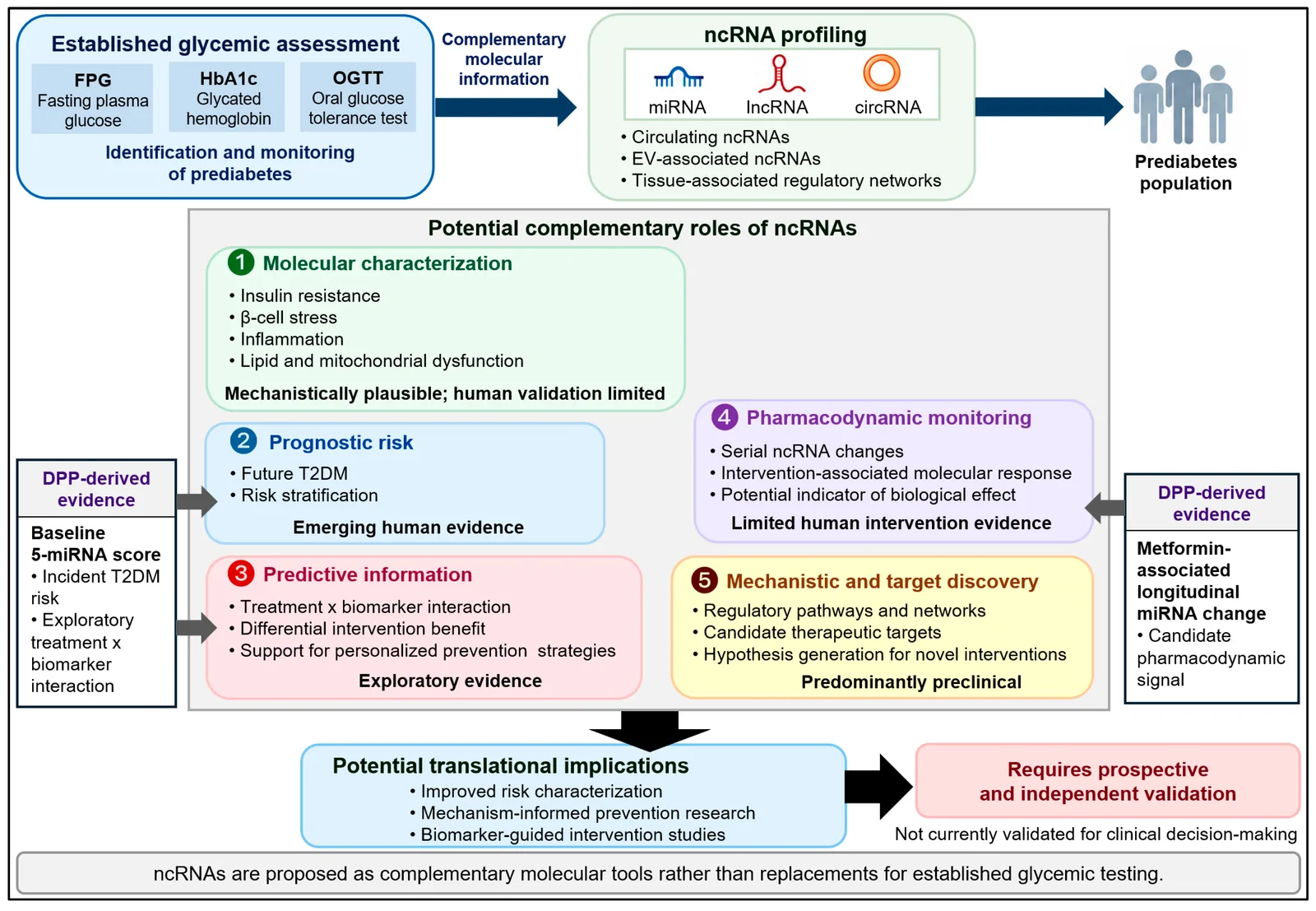
Potential complementary roles of non-coding RNAs across the prediabetes prevention continuum. Prediabetes is identified and monitored using established clinical and glycemic measures, including fasting plasma glucose (FPG), glycated hemoglobin (HbA1c), and the oral glucose tolerance test (OGTT). Non-coding RNA (ncRNA) profiling is proposed as a complementary molecular layer rather than a replacement for these established approaches. Potential applications can be considered across several distinct biomarker and mechanistic functions. First, ncRNA profiles may contribute to molecular characterization by providing information related to insulin resistance, β-cell stress, inflammation, lipid dysregulation, and other regulatory processes, although ncRNA-based molecular subtyping of human prediabetes remains unvalidated. Second, baseline circulating ncRNA profiles may provide prognostic information regarding future progression to type 2 diabetes mellitus (T2DM). Third, baseline biomarkers showing a treatment-by-biomarker interaction may have predictive potential for differential benefit from preventive interventions; current evidence is limited and largely exploratory. Fourth, serial changes in circulating ncRNAs after lifestyle or pharmacological intervention may be evaluated as pharmacodynamic signals of treatment-associated molecular responses. Finally, mechanistic ncRNA studies may identify candidate regulatory pathways and therapeutic targets, although therapeutic translation remains predominantly preclinical. Evidence supporting these roles varies substantially across applications, from direct human prediabetes cohorts to related metabolic conditions and experimental models. Accordingly, all ncRNA-based applications shown in this framework remain investigational and require prospective, independent validation before clinical implementation.

The evidence base in prediabetes is uneven across these biomarker functions. Most published human studies are cross-sectional and evaluate whether circulating miRNAs differ between normoglycemia, prediabetes, and established T2DM [21,22,23]. A smaller number of prospective studies have examined progression to incident diabetes [24,25], and only limited randomized intervention data are available for predictive or pharmacodynamic applications [25,26]. The following subsections therefore evaluate circulating ncRNAs according to the information they may potentially add beyond glycemic classification, while emphasizing the distinction between proof-of-concept molecular associations and clinically actionable biomarker performance. Figure 4 summarizes this translational framework, distinguishing established glycemic classification from the potential complementary roles of ncRNAs in molecular characterization, prognostic risk stratification, treatment prediction, pharmacodynamic monitoring, and mechanistic target discovery.

### 5.1. Diagnostic and Discriminative Evidence: Clinical Relevance Beyond Glycemic Testing

A number of circulating miRNAs have shown differences between individuals with normoglycemia, prediabetes, and T2DM, supporting proof-of-concept that ncRNA profiles can discriminate between glycemic states. Reported candidates include miR-126, miR-126-3p, miR-186-5p, miR-21, miR-93-5p, miR-652-3p, and let-7i-5p, although the magnitude and direction of associations vary across study populations, biospecimens, and analytical platforms [19,20,21,22,23]. These findings indicate that circulating miRNA profiles are responsive to early metabolic dysregulation, but cross-sectional differences alone do not establish that ncRNA testing provides clinically meaningful information beyond established glycemic measurements. Representative human studies are summarized in Table 1, with emphasis on study design, metabolic context, biospecimen, biomarker interpretation, and the directness of evidence for prediabetes. The table separates direct human prediabetes evidence from supportive findings derived from related metabolic states and distinguishes cross-sectional discrimination from longitudinal prognostic evidence. This distinction is important because strong discrimination between existing glycemic categories should not be interpreted as evidence of future progression risk or treatment-predictive utility.

Several studies have reported moderate discriminative performance for individual circulating miRNAs. In a two-stage study, plasma EV-associated miR-186-5p distinguished prediabetes from normoglycemia in the validation cohort with an area under the receiver operating characteristic curve (AUC) of 0.745 [20]. Whole-blood miR-126-3p showed an AUC of 0.76 for distinguishing prediabetes from normal glucose tolerance in a population-based cohort [22], whereas plasma miR-21 showed an AUC of 0.77 in a smaller case–control study [23]. These findings demonstrate molecular discrimination but should be interpreted cautiously because comparable glycemic classification can already be achieved using established clinical tests. A more relevant translational question is whether circulating ncRNAs provide incremental information beyond FPG, HbA1c, OGTT, or established clinical risk factors. Evidence addressing this question remains limited. Although some studies report associations after adjustment for metabolic covariates, few have formally tested whether adding ncRNA measurements improves model discrimination, calibration, reclassification, or clinical decision-making beyond conventional risk assessment. Likewise, external validation across independent populations remains uncommon. Methodological heterogeneity further limits interpretation. Reported ncRNA levels can differ according to plasma versus serum sampling, whole-blood versus EV-enriched fractions, RNA isolation procedures, normalization strategies, hemolysis, and analytical platforms such as RT-qPCR and small RNA sequencing [60,82,83]. Population differences in age, obesity, ethnicity, medication exposure, and comorbidity may also contribute to inconsistent replication of specific candidates.

Accordingly, the current evidence supports circulating ncRNAs as candidate molecular discriminators of dysglycemic states, but not as replacements for established glycemic testing. Their future clinical value will depend on demonstrating that they provide information that conventional measurements do not already capture—particularly with respect to future progression risk, biological heterogeneity, or intervention-associated molecular responses. These higher-value applications are considered in the subsequent subsections.

**Table 1:** Representative human studies evaluating circulating miRNAs in prediabetes and related metabolic states: diagnostic and prognostic evidence.

miRNA	Direction/Association	Study Design, Cohort, and Metabolic Context (*n*; Criteria)	Specimen	Main Finding/ Biomarker Interpretation	Evidence Context	Ref.
miR-126	↓	Cross-sectional baseline comparison with 6-month intervention follow-up; *n* = 455: healthy controls 138, IGT 82, IFG 75, newly diagnosed T2DM 160; IGT/IFG defined by WHO criteria	Serum	Lower in IGT/IFG and newly diagnosed T2DM than in controls; supports cross-sectional discrimination of dysglycemia. Levels increased after lifestyle intervention in IGT/IFG participants	Direct human prediabetes—cross-sectional with intervention follow-up	[21]
miR-126-3p	↑	Cross-sectional population study; *n* = 1273: NGT 972, prediabetes 207, T2DM 94; glycemic status determined by OGTT	Whole blood	Higher in prediabetes than NGT; discriminated prediabetes from NGT (AUC 0.760), exceeding HbA1c in this cohort	Direct human prediabetes—large population-based cross-sectional cohort	[22]
miR-186-5p	↓	Two-stage study: screening *n* = 60 (20/group) and independent validation *n* = 150 (50 normoglycemia, 50 prediabetes, 50 T2DM); ADA criteria	Plasma EV	Reduced in prediabetes and T2DM; independently associated with prediabetes in the validation cohort; exploratory discrimination of prediabetes from normoglycemia (AUC 0.745)	Direct human prediabetes—two-stage study with independent validation	[20]
miR-21	↑	Cross-sectional study; *n* = 82: controls 29, prediabetes 29, T2DM 24; ADA-based glycemic criteria, participants with familial T2DM susceptibility	Plasma	Higher in prediabetes and T2DM than controls; discriminated prediabetes from controls (AUC 0.77). Supports diagnostic/discriminative, but not treatment-predictive, utility	Direct human prediabetes—small exploratory cross-sectional cohort	[23]
miR-93-5p	↑	Exploratory cross-sectional study in obese women; *n* = 64: NGT 44, IFG 20	Plasma	Higher in IFG than NGT and correlated with fasting glucose; exploratory prediabetes-associated marker	Direct human prediabetes—small exploratory cross-sectional cohort	[19]
miR-652-3p	↑	Same exploratory cohort; *n* = 64: NGT 44, IFG 20	Plasma	Higher in IFG than NGT; exploratory prediabetes-associated marker	Direct human prediabetes—small exploratory cross-sectional cohort	[19]
let-7i-5p	↑	Same exploratory cohort; *n* = 64: NGT 44, IFG 20	Plasma	Higher in IFG than NGT and correlated with fasting glucose; supported by complementary mechanistic experiments	Direct human prediabetes—small exploratory cross-sectional cohort	[19]
miR-192	↑ in T2DM/incident T2DM; not clearly ↑ in IFG	Cross-sectional validation: NFG 43, IFG 43, T2DM 43; prospective cohort *n* = 213 diabetes-free participants, 35 incident T2DM over 6 years	Serum	Associated with incident T2DM independently of conventional risk factors; supports prognostic potential, but not a prediabetes-specific diagnostic marker	Supportive human evidence—prospective incident T2DM cohort; not prediabetes-specific	[84]
miR-122	↑	Prospective population-based cohort; *n* = 810; up to 15 years of follow-up	Serum	Higher circulating levels associated with future metabolic syndrome and T2DM; supportive prognostic evidence but not validated specifically in a prediabetes cohort	Supportive human evidence—large prospective population cohort; not prediabetes-specific	[85]
miR-107	↑	Case–control study; *n* = 107: T2DM 53, non-diabetic controls 54; controls had neither diabetes nor prediabetes	Serum	Higher in T2DM and associated with BMI, dyslipidemia, and HOMA-IR; supportive evidence for metabolic dysregulation without prediabetes-specific validation	Supportive human evidence—T2DM case–control cohort; no prediabetes-specific validation	[86]
miR-491-5p, miR-1307-3p, miR-298	↑ in progressors	5-year prospective observational study; *n* = 42 individuals with prediabetes at baseline; 24 progressed to T2DM and 18 remained non-diabetic	Serum	Higher baseline levels in future progressors; AUCs 0.94, 0.88, and 0.84, respectively; supports exploratory prognostic potential for progression from prediabetes to T2DM	Direct human prediabetes—5-year prospective exploratory cohort	[24]
5-miRNA score: miR-144, miR-186, miR-203a, miR-205, miR-206	↑ score in progressors	Secondary analysis of randomized DPP cohort; *n* = 1000 adults with prediabetes	Plasma	Baseline score associated with incident T2DM after adjustment for conventional risk factors; exploratory treatment-by-biomarker interaction reported across randomized intervention groups	Direct human prediabetes—large prospective randomized-trial cohort (DPP); prognostic with exploratory predictive evidence	[25]

Evidence context indicates the directness and design of the human evidence relevant to prediabetes rather than a formal evidence-grading system. “Direct human prediabetes” denotes studies enrolling participants with prediabetes according to established glycemic criteria, whereas “supportive human evidence” refers to studies conducted in related metabolic populations or incident T2DM cohorts without dedicated prediabetes-specific validation. Prospective, intervention, and independent-validation features are specified where applicable. ADA, American Diabetes Association; AUC, area under the receiver operating characteristic curve; BMI, body mass index; EV, extracellular vesicle; HOMA-IR, homeostatic model assessment of insulin resistance; IFG, impaired fasting glucose; IGT, impaired glucose tolerance; NGT, normal glucose tolerance; OGTT, oral glucose tolerance test; T2DM, type 2 diabetes mellitus; WHO, World Health Organization; ↑, increased; ↓, decreased.

### 5.2. Prognostic Risk Stratification for Progression to T2DM

From a clinical perspective, the potential value of circulating ncRNAs in prediabetes may be greater for prognostic risk stratification than for cross-sectional discrimination. Prediabetes is heterogeneous in its natural history: some individuals progress to T2DM, whereas others remain stable or return to normoglycemia [2,4,5]. Established glycemic and clinical risk factors already provide useful prognostic information, but circulating ncRNAs may potentially capture additional molecular features associated with future metabolic deterioration [24,25]. Accordingly, prospective studies are particularly important for determining whether baseline ncRNA profiles are associated with subsequent progression rather than merely reflecting the current glycemic state.

Direct human evidence remains limited but includes several prospective studies. In a 5-year observational study of individuals with prediabetes, baseline circulating miR-491-5p, miR-1307-3p, and miR-298 were higher in participants who subsequently progressed to T2DM than in those who remained non-diabetic [24]. Individual miRNAs showed relatively high exploratory discrimination for future progression, with reported AUCs ranging from 0.84 to 0.94, and the combined three-miRNA model showed further improvement in discrimination. However, the cohort was small, and the findings have not been independently validated. These results therefore provide proof-of-concept that circulating miRNA profiles may contain prognostic information, but they do not yet establish a clinically generalizable risk model. Stronger cohort-level evidence is provided by a secondary analysis of 1000 participants from the Diabetes Prevention Program (DPP), in which a baseline 5-miRNA score comprising miR-144, miR-186, miR-203a, miR-205, and miR-206 was associated with incident T2DM [25]. Importantly, the association remained significant after adjustment for conventional risk factors, including body mass index, fasting glucose, and HbA1c, suggesting that the composite miRNA profile may capture molecular information not fully represented by standard clinical variables. Nevertheless, the incremental clinical value of this score has not yet been established through independent external validation, formal reclassification analyses, or prospective implementation in routine care. Additional prospective studies conducted outside dedicated prediabetes cohorts provide supportive evidence. Circulating miR-192 and miR-122 have been associated with future T2DM or related metabolic outcomes in population-based studies [84,85]. These findings strengthen the general concept that circulating miRNAs may precede overt diabetes, but they should not be interpreted as direct validation of prediabetes-specific prognostic markers.

Taken together, current evidence supports prognostic risk stratification as one of the more plausible future clinical applications of ncRNA profiling in prediabetes. However, the central translational question is not whether an ncRNA is statistically associated with future diabetes, but whether it improves prediction beyond established clinical models in a reproducible and clinically meaningful manner. Future studies should therefore evaluate discrimination, calibration, reclassification, and decision-analytic performance after adding ncRNA profiles to conventional risk factors, with external validation across diverse populations. The DPP 5-miRNA score is particularly relevant because the same cohort also provides exploratory evidence of a treatment-by-biomarker interaction; this distinct predictive application is discussed separately in the intervention section.

### 5.3. EV-Associated ncRNAs as Potentially Tissue-Informative Molecular Signals

Extracellular vesicle (EV)-associated ncRNAs represent a distinct subset of circulating molecular signals with potential relevance to metabolic disease. EVs can protect RNA cargo from extracellular degradation and can undergo regulated release and cargo selection in response to cellular stress [17,60]. These properties have generated interest in whether EV-associated ncRNAs may provide biologically enriched information compared with total circulating RNA measurements. However, the presence of an ncRNA within circulating EVs does not by itself establish the tissue or cell type from which the vesicle originated. EV cargo composition is influenced by RNA-binding proteins, sequence-dependent sorting mechanisms, vesicle biogenesis, and the physiological state of the releasing cell [17]. Metabolic stress may therefore alter both EV release and ncRNA cargo composition, raising the possibility that circulating EV profiles contain molecular information related to insulin resistance, β-cell stress, inflammation, or other components of early metabolic dysfunction. Nevertheless, this potential should be described as tissue-informative rather than tissue-specific, because circulating EV populations are heterogeneous and usually cannot be assigned to a defined tissue without additional molecular or experimental validation. Plasma EV-associated miR-186-5p provides a representative example linking a circulating EV signal with both human prediabetes and a plausible metabolic mechanism. In a two-stage human study, EV-associated miR-186-5p was reduced in individuals with prediabetes, while complementary experimental studies linked reduced miR-186-5p to IFN-α/JAK–STAT signaling, mitochondrial oxidative stress, and impaired β-cell function [20]. This combination of clinical association and mechanistic support illustrates the potential value of EV-associated ncRNAs for connecting circulating molecular signals with biologically relevant pathways. However, the circulating EV signal does not establish pancreatic β-cells as the exclusive source of miR-186-5p, nor does it demonstrate that EV measurement provides incremental clinical value beyond established metabolic markers.

EV-associated ncRNAs may also participate in intercellular communication. Experimental studies indicate that EVs released from one cell or tissue can be taken up by recipient cells and modify gene expression through transferred RNA cargo [17]. Such findings provide a mechanistic rationale for considering EV-associated ncRNAs not only as circulating indicators but also as potential mediators of metabolic signaling. However, direct evidence demonstrating functionally important EV-mediated ncRNA communication during human prediabetes remains limited, and this concept should therefore be regarded as mechanistically plausible rather than clinically established. Translation of EV-associated ncRNA profiles into reproducible biomarkers is further constrained by methodological variability. Ultracentrifugation, size-exclusion chromatography, precipitation-based approaches, and other isolation procedures recover EV populations that differ in purity, size distribution, and co-isolated non-vesicular material [60]. Pre-analytical variables, EV characterization, RNA extraction, normalization, and analytical platform can consequently influence measured ncRNA profiles. Such methodological differences can alter not only the absolute abundance of individual ncRNAs but also the composition and relative ranking of candidate biomarker signatures. Consequently, diagnostic or prognostic thresholds derived using one EV isolation and analytical workflow cannot be assumed to be transferable across laboratories, platforms, or patient cohorts without technical harmonization and independent cross-platform validation. Adherence to standardized reporting frameworks such as Minimal Information for Studies of Extracellular Vesicles 2023 (MISEV2023) and prospective validation in well-characterized prediabetes cohorts will be essential for determining whether EV-associated ncRNAs provide reproducible information beyond total circulating ncRNA measurements and established clinical variables [60]. Thus, EV-associated ncRNAs may offer a biologically informative layer within circulating biomarker research, but their principal potential currently lies in linking accessible molecular signals with underlying metabolic processes rather than in providing tissue-specific or clinically validated diagnostic information. Whether EV-based profiling improves prognostic risk stratification, molecular phenotyping, or intervention monitoring beyond simpler circulating RNA measurements remains an important question for future studies.

Taken together, current human evidence supports three progressively distinct levels of potential information from circulating ncRNAs in prediabetes: discrimination of existing glycemic states, prognostic association with future T2DM, and biologically informative circulating signals that may reflect underlying metabolic processes. A further possibility is that ncRNA profiles could eventually contribute to molecular phenotyping of heterogeneous prediabetes by distinguishing individuals in whom insulin resistance, β-cell stress, inflammation, or other metabolic pathways predominate. However, direct evidence for ncRNA-based molecular subtyping in human prediabetes is currently insufficient. This application should therefore be regarded as a testable future direction rather than an established biomarker function.

## 6. The Diabetes Prevention Program as a Translational Framework for ncRNA Biomarkers

The Diabetes Prevention Program (DPP) provides a particularly informative setting for evaluating the potential translational roles of ncRNA biomarkers in prediabetes. Unlike most cross-sectional biomarker studies, the DPP enrolled individuals at high risk for T2DM and randomly assigned them to intensive lifestyle intervention, metformin, or placebo, with subsequent longitudinal assessment of diabetes incidence [6]. This design makes it possible to distinguish several biomarker functions that are often conflated in observational ncRNA studies: baseline markers associated with future disease risk, baseline markers associated with differential benefit across interventions, and molecular changes occurring after treatment exposure. Two secondary analyses of the DPP are especially relevant to this distinction. Flowers et al. evaluated baseline circulating miRNA profiles in relation to incident T2DM and randomized intervention assignment [25], whereas Lewis et al. examined longitudinal changes in circulating miRNAs after metformin exposure compared with placebo [26]. Together, these studies provide a rare opportunity to consider prognostic, exploratory predictive, and candidate pharmacodynamic information within the same prevention-trial framework. As summarized conceptually in Figure 4, these functions represent distinct translational questions and should not be interpreted interchangeably. The DPP evidence remains preliminary from a biomarker-development perspective. Both analyses were secondary studies, and neither the baseline miRNA score nor the treatment-associated miRNA changes have undergone independent prospective validation for clinical use. Nevertheless, the DPP provides a useful proof-of-concept model for how ncRNA research can move beyond cross-sectional discrimination toward questions that are more relevant to diabetes prevention: who is most likely to progress, whether baseline molecular profiles modify the relative benefit of specific interventions, and whether treatment exposure is accompanied by reproducible molecular changes.

### 6.1. From Prognostic Risk to Exploratory Predictive Information: The DPP 5-miRNA Score

As discussed in Section 5.2, Flowers et al. identified a baseline 5-miRNA score comprising miR-144, miR-186, miR-203a, miR-205, and miR-206 that was associated with incident T2DM among 1000 DPP participants [25]. Because this association was evaluated prospectively and remained significant after adjustment for conventional clinical risk factors, the score provides evidence for a prognostic biomarker function. Importantly, however, the randomized design of the DPP also allowed the investigators to examine a separate question: whether the association between the baseline miRNA score and diabetes risk differed according to intervention assignment. The reported treatment-by-biomarker interaction raises the possibility that baseline miRNA profiles may contain information related to differential benefit from lifestyle intervention, metformin, or placebo [25]. In biomarker terminology, this represents exploratory predictive evidence, which is conceptually distinct from prognosis. A prognostic biomarker identifies individuals at higher or lower risk of an outcome regardless of treatment, whereas a predictive biomarker identifies variation in treatment benefit according to baseline biomarker status. The distinction is important because a marker can be strongly associated with future T2DM without necessarily informing treatment selection.

The DPP analysis is therefore notable because it extends ncRNA evaluation beyond disease-risk association toward the possibility of intervention-specific information. However, this interpretation should remain cautious. The treatment-by-biomarker finding derives from a single secondary analysis, has not been independently replicated, and was not prospectively prespecified for clinical treatment selection. Moreover, the extent to which the miRNA score improves decision-making beyond established clinical characteristics has not been established. Accordingly, the 5-miRNA score should be considered a candidate prognostic biomarker with exploratory predictive potential, rather than a validated tool for selecting preventive therapy. This distinction has broader implications for ncRNA research in prediabetes. Future studies should prespecify treatment-by-biomarker interaction analyses, validate candidate signatures in independent randomized or prospective cohorts, and determine whether molecular profiling meaningfully improves treatment allocation beyond readily available clinical variables. If such evidence emerges, baseline ncRNA profiles could eventually contribute to a more individualized prevention framework; at present, however, their predictive role remains investigational.

### 6.2. Metformin-Associated miRNA Dynamics as Candidate Pharmacodynamic Signals

Whereas baseline miRNA profiles may provide prognostic or exploratory predictive information, longitudinal changes in ncRNA abundance after treatment address a different biomarker question. Such post-intervention changes are most appropriately considered candidate pharmacodynamic signals, provided that they reproducibly reflect treatment-associated molecular responses. The DPP provides direct human evidence for this application through a secondary analysis comparing circulating miRNA profiles after metformin treatment with those observed in the placebo group [26].

Lewis et al. evaluated circulating miRNA changes over two years in DPP participants assigned to metformin or placebo [26]. Six miRNAs—let-7c-5p, miR-151a-3p, miR-17-5p, miR-20b-5p, miR-29b-3p, and miR-93-5p—were increased after metformin relative to placebo, whereas miR-130b-3p, miR-22-3p, miR-222-3p, miR-320a-3p, miR-320c, and miR-92a-3p were decreased [26]. Because these differences were observed within a randomized prediabetes intervention cohort, they provide relatively direct evidence that metformin exposure is associated with a distinct circulating miRNA response. The biological interpretation of these changes remains less certain. Several of the identified miRNAs have been implicated in insulin signaling, inflammation, lipid metabolism, or other metabolic pathways, suggesting that the observed profile may reflect molecular effects of metformin beyond changes in glycemia alone. However, the study does not establish that the direction or magnitude of individual miRNA changes quantitatively tracks therapeutic efficacy, mediates the clinical benefit of metformin, or provides information that is independent of conventional treatment-response measures. This distinction is important for biomarker classification. A pharmacodynamic biomarker reflects a biological response occurring after exposure to an intervention, whereas a predictive biomarker is measured before treatment and identifies differential benefit across interventions. Accordingly, the longitudinal miRNA changes reported by Lewis et al. should not be interpreted as evidence that these miRNAs can guide treatment selection. Rather, they provide proof-of-concept that serial ncRNA profiling can detect treatment-associated molecular changes in individuals with prediabetes.

Further studies should determine whether these miRNA responses are reproducible across independent cohorts, whether they show consistent temporal relationships with metabolic improvement, and whether they provide incremental information beyond changes in glucose, HbA1c, body weight, or insulin-resistance indices. As illustrated in Figure 4, such evidence would be required before serial ncRNA measurements could be considered clinically meaningful pharmacodynamic biomarkers rather than treatment-associated molecular signals.

Taken together, the DPP analyses illustrate three conceptually distinct ncRNA biomarker functions within a single prevention-trial framework: prognostic association with future T2DM, exploratory prediction of differential intervention benefit, and treatment-associated pharmacodynamic change. Although none is currently validated for clinical implementation, this framework provides a useful model for future ncRNA studies designed to move beyond cross-sectional discrimination toward clinically relevant prevention questions.

### 6.3. From ncRNA Information to Potential Clinical Decisions

For ncRNA profiling to have clinical utility, molecular information would need to alter a prevention decision that would not be made from conventional risk factors alone. Current clinical recommendations advise at least annual monitoring for individuals with prediabetes, with testing frequency modified according to individual risk, referral of adults with overweight or obesity at high risk of T2DM to structured intensive lifestyle intervention, and consideration of metformin in selected high-risk individuals [87]. Within this framework, a prospectively validated prognostic ncRNA signature that meaningfully reclassifies progression risk beyond FPG, HbA1c, adiposity, age, and other established variables could be tested as a basis for more intensive behavioral support and more frequent metabolic reassessment than the minimum annual schedule. A predictive ncRNA signature would serve a different purpose: only a reproducible treatment-by-biomarker interaction could justify using ncRNA information to refine whether metformin is added to lifestyle intervention among otherwise eligible high-risk individuals. Serial ncRNA changes could, in turn, be evaluated as adjunct pharmacodynamic markers of biological response to intervention, but they should complement rather than replace established measures such as glycemia, body weight, and other clinical outcomes. None of these ncRNA-guided strategies is currently validated. Future prevention trials should therefore test prespecified ncRNA-guided action algorithms and determine whether they improve clinical decision-making or patient-relevant outcomes compared with conventional risk assessment alone.

## 7. ncRNA Dynamics During Other Lifestyle and Pharmacological Interventions

Beyond the DPP, a growing number of studies have examined ncRNA changes after lifestyle or pharmacological interventions in prediabetes and related metabolic conditions. These studies are relevant because they test whether ncRNA profiles are responsive to interventions that modify metabolic risk. However, their interpretation requires caution. Most studies have not demonstrated that ncRNA changes mediate clinical benefit, predict differential treatment response, or provide information beyond established metabolic outcomes. Accordingly, post-intervention ncRNA changes are considered here primarily as candidate pharmacodynamic or intervention-associated molecular signals, with the strength of evidence determined by study design and clinical context. The available evidence is heterogeneous. Direct randomized human data in prediabetes are limited largely to DPP-derived studies, whereas lifestyle-intervention findings come from prediabetes and related cardiometabolic populations, and evidence for several pharmacological agents derives mainly from established T2DM or preclinical models. Table 2 summarizes representative intervention studies and distinguishes direct human prediabetes evidence from supportive human and preclinical findings. Importantly, treatment-associated ncRNA changes should not be interpreted as predictive biomarkers unless a baseline treatment-by-biomarker interaction has been demonstrated.

### 7.1. Lifestyle Interventions: Exercise, Diet, and Weight Loss

Lifestyle modification remains the primary intervention for reducing the risk of progression from prediabetes to T2DM [6,7]. Exercise, dietary modification, and weight loss improve insulin sensitivity, body composition, and glycemic control, and several studies have reported concurrent changes in circulating miRNA profiles [21,88,91]. These observations raise the possibility that serial ncRNA measurements may provide molecular information about biological responses to lifestyle intervention. However, the available evidence remains heterogeneous, and changes in ncRNA abundance should not be assumed to represent beneficial treatment effects without demonstrating a consistent relationship with clinical or metabolic improvement.

miR-126 is one of the most frequently investigated circulating miRNAs in this context. In individuals with impaired glucose tolerance (IGT) or impaired fasting glucose (IFG), circulating miR-126 levels were lower than those in healthy controls and increased after six months of diet control and physical exercise [21]. This finding provides direct human evidence that circulating miR-126 is responsive to lifestyle intervention in a prediabetic population. However, the study was not designed to establish whether the magnitude of miR-126 change predicted the degree of metabolic improvement or provided information beyond conventional glycemic measurements. Intervention-associated changes in miR-126 have also been reported in related cardiometabolic populations, but the direction of change has not been consistent. In patients with heart failure with preserved ejection fraction, circulating miR-126 increased after exercise rehabilitation [91], whereas a six-week combined exercise-and-diet intervention in obese adolescents was associated with a decrease in circulating miR-126 despite improvement in glucolipid metabolism and microvascular endothelial function [88]. These divergent findings indicate that circulating miR-126 responses are highly context-dependent and may vary according to baseline metabolic state, intervention type and duration, biospecimen, sampling time, and analytical methodology. This heterogeneity is particularly important for pharmacodynamic interpretation. A useful pharmacodynamic biomarker should show a reproducible relationship with intervention exposure or biological response. At present, the direction of circulating miR-126 change cannot be interpreted consistently as favorable or unfavorable, and no validated threshold has been established for treatment monitoring. Therefore, miR-126 should currently be regarded as an intervention-responsive molecular signal rather than a validated pharmacodynamic biomarker.

More broadly, lifestyle interventions have been associated with changes in multiple circulating miRNAs in obesity, cardiovascular disease, and other insulin-resistant states [88,91,93]. These studies provide supportive evidence that ncRNA profiles can respond dynamically to metabolic intervention, but they do not establish that the observed changes are specific to prediabetes or that they add clinically meaningful information beyond body weight, glucose, HbA1c, or measures of insulin resistance. Future studies should therefore incorporate standardized serial sampling, prespecified ncRNA endpoints, and parallel assessment of conventional metabolic outcomes to determine whether ncRNA dynamics provide reproducible and independent information about biological responses to lifestyle intervention.

### 7.2. Pharmacological Interventions Beyond DPP Metformin

Beyond the randomized DPP evidence for metformin, pharmacological modulation of ncRNA profiles has been reported in several related metabolic and cardiometabolic settings. These studies provide supportive evidence that drug exposure can alter circulating, EV-associated, or tissue ncRNA profiles, but their relevance to prediabetes varies substantially according to population, study design, and biological context. Importantly, treatment-associated ncRNA changes observed in established T2DM, polycystic ovary syndrome (PCOS), or experimental models should not be extrapolated directly to prediabetes without dedicated validation. Additional evidence for metformin-associated miRNA modulation comes from women with PCOS, a condition characterized by insulin resistance and metabolic dysfunction. In a randomized intervention study, circulating miR-122 and miR-29a decreased after metformin treatment relative to the metformin-plus-oral-contraceptive and oral-contraceptive groups, whereas miR-223 decreased in the combined metformin-plus-oral-contraceptive group [89]. These findings support the responsiveness of circulating miRNAs to metformin-containing interventions in an insulin-resistant population. However, because the study population, hormonal context, and concomitant treatment differ from those of prediabetes, the results should be interpreted as supportive pharmacodynamic evidence rather than as direct validation of prediabetes-specific biomarkers.

Glucagon-like peptide-1 receptor agonists (GLP-1RAs) have also been associated with changes in circulating miRNA profiles. In a small single-arm study of patients with established T2DM receiving liraglutide in addition to metformin, serum levels of miR-27b, miR-130a, and miR-210 increased after four months of treatment [90]. These miRNAs have been linked to endothelial and cardiometabolic pathways, suggesting that GLP-1RA exposure may be accompanied by measurable changes in regulatory RNA profiles. However, the absence of a randomized control group and the use of an established T2DM population limit causal and prediabetes-specific interpretation. The observed changes should therefore be regarded as candidate treatment-associated molecular signals rather than validated pharmacodynamic biomarkers.

Preclinical studies provide additional mechanistic support for drug-associated ncRNA modulation. In an ovariectomized rat model of T2DM, liraglutide altered exosomal miRNA profiles linked by pathway analysis to insulin secretion and insulin-signaling processes [94]. Likewise, sodium-glucose cotransporter 2 (SGLT2) inhibition in a rat model of diabetic cardiomyopathy was associated with suppression of miR-30d and modulation of the miR-30d/KLF9/VEGFA axis, together with changes in cardiac autophagy and function [92]. These studies demonstrate that pharmacological interventions can influence ncRNA-related pathways in vivo, but they do not establish circulating biomarkers or treatment-response markers in human prediabetes.

Taken together, current pharmacological evidence outside the DPP supports the broader concept that ncRNA profiles are responsive to metabolic drug exposure, but the translational evidence remains limited. At present, these findings are most appropriately interpreted as supportive human or preclinical pharmacodynamic evidence, not as proof that ncRNA measurements can guide treatment selection or monitor therapeutic efficacy in prediabetes. Future studies should prioritize prospective prediabetes cohorts, standardized pre- and post-treatment sampling, appropriate control groups, and direct comparison with established clinical measures to determine whether pharmacologically induced ncRNA changes provide reproducible and clinically meaningful information.

## 8. Methodological and Clinical Translation Challenges

Translation of ncRNA profiling into clinically useful tools for prediabetes will require more than the identification of statistically significant differences between metabolic groups. Candidate biomarkers must demonstrate analytical reproducibility, biological robustness, independent clinical validity, and, ultimately, clinically meaningful information beyond established glycemic and clinical risk measures. These requirements remain major barriers across circulating miRNA, lncRNA, circRNA, and extracellular vesicle (EV)-associated biomarker studies.

Pre-analytical variability represents a major source of inconsistency in circulating ncRNA measurements. Serum and plasma should not be considered interchangeable matrices because coagulation, platelet activation, and residual cellular components can alter the abundance of specific circulating miRNAs. Blood collection conditions, time to processing, centrifugation protocols, storage temperature, and repeated freeze–thaw cycles can further affect measured ncRNA profiles. Hemolysis is particularly important because even limited erythrocyte disruption can release highly abundant intracellular miRNAs into serum or plasma and thereby generate apparent biomarker differences that do not reflect the underlying metabolic state. Recent studies have therefore emphasized prespecified quality-control procedures for hemolysis and platelet contamination together with standardized sample processing [82,95]. RNA extraction introduces an additional source of variability because differences in extraction chemistry, input volume, carrier RNA, and recovery efficiency can disproportionately affect low-abundance circulating RNAs. For EV-associated ncRNAs, methodological heterogeneity is further amplified by differences among ultracentrifugation, size-exclusion chromatography, precipitation-based methods, and other isolation approaches, which recover vesicle populations with different purity and varying amounts of co-isolated non-vesicular RNA. Standardized reporting of pre-analytical conditions and adherence to MISEV2023 recommendations for EV isolation and characterization are therefore essential for improving cross-study reproducibility [60,96].

Analytical platform and normalization strategy provide an additional layer of variability. RT-qPCR is well suited to sensitive, targeted measurement of predefined ncRNAs and to validation studies, but quantitative results depend on reverse-transcription efficiency, primer or probe design, amplification performance, and the choice of endogenous or exogenous reference controls. Circulating ncRNAs lack a universally stable endogenous reference across biological and disease contexts, making normalization a persistent source of between-study variability. Small RNA sequencing, in contrast, enables broader and less hypothesis-driven profiling but is susceptible to library-preparation and adapter-ligation biases, sequencing-depth effects, and bioinformatic normalization choices; importantly, different library-preparation protocols can yield substantially different representations of the same circulating miRNA pool [83,97]. Consequently, biomarker effect sizes, candidate rankings, and diagnostic or prognostic thresholds generated on one platform cannot be assumed to transfer directly to another. Discovery by small RNA sequencing should therefore be followed by orthogonal technical validation, typically using RT-qPCR or an equivalent quantitative platform, together with explicit reporting of normalization and quality-control procedures. Adoption of platform-specific reporting standards, including MISEV2023 for EV studies and updated Minimum Information for Publication of Quantitative Real-Time PCR Experiments (MIQE) recommendations for RT-qPCR, should improve analytical reproducibility and facilitate cross-cohort validation [60,98].

For longitudinal intervention studies, these considerations are particularly important because apparent treatment-associated ncRNA changes may reflect batch effects or platform-related variation unless paired samples are processed and analyzed under rigorously standardized conditions [82,83]. Reproducible normalization, rigorous quality control, and cross-platform validation will therefore be necessary before circulating ncRNA signatures can be reliably compared across cohorts or translated into clinically applicable cutoffs.

Biological sex represents an additional source of heterogeneity that should be considered in ncRNA biomarker development. Sex differences in insulin sensitivity, adipose tissue distribution and function, inflammatory signaling, and metabolic disease progression are well established and vary further across life stages such as menopause [99,100]. Sex hormones, sex-chromosome-linked mechanisms, and sex-dependent metabolic and immune environments can in turn influence ncRNA expression and regulatory activity [101]. Consistent with this concept, a recent exploratory plasma small-RNA sequencing study including individuals with diabetes, prediabetes, and normoglycemia identified significant sex-associated differences in circulating miRNA profiles [102]. However, the prediabetes subgroup in that study was very small, and robust evidence for prediabetes-specific sex differences in circulating ncRNA biomarkers remains limited [102]. Pooled analyses that ignore such heterogeneity may obscure true associations or yield biomarker signatures with sex-dependent calibration or diagnostic performance. Future discovery and validation studies should therefore prespecify sex-stratified analyses and ncRNA-by-sex interaction testing, and should evaluate discrimination, calibration, and clinically relevant thresholds separately by sex where adequately powered. In women, menopausal status and exogenous hormone exposure should also be considered when relevant.

Clinical reproducibility presents an equally important challenge. Many human ncRNA studies relevant to prediabetes remain relatively small, cross-sectional, or insufficiently externally validated [103]. Prediabetes itself is biologically heterogeneous, and ncRNA profiles may be influenced by ethnicity, age, adiposity, diet, medication use, comorbidities, inflammatory status, and other environmental or metabolic factors. Consequently, a signature identified in one population may not reproduce in another. Larger multicenter and multi-ethnic longitudinal cohorts, together with independent external validation, will be required to distinguish stable disease-associated signals from population- or context-dependent variation. Importantly, replication alone is not sufficient to establish clinical utility. For diagnostic applications, ncRNA profiles must demonstrate whether they provide meaningful information beyond FPG, HbA1c, and OGTT rather than simply reproducing existing glycemic classification. For prognostic applications, candidate signatures should be evaluated against established clinical risk models using measures of discrimination, calibration, reclassification, and, where appropriate, decision-analytic performance. Predictive biomarkers require prospectively evaluated treatment-by-biomarker interactions, whereas pharmacodynamic biomarkers require reproducible temporal relationships between intervention exposure, molecular change, and relevant biological or clinical responses. These distinctions are essential because cross-sectional discrimination, prospective association, treatment prediction, and intervention-associated molecular change represent different levels of biomarker evidence.

Thus, the principal translational challenge is not simply to identify additional ncRNAs associated with prediabetes, but to determine whether standardized ncRNA measurements provide reproducible and clinically informative molecular information beyond conventional assessment. Future studies should therefore prioritize prespecified biomarker functions, harmonized biospecimen and analytical protocols, cross-platform technical validation, prospective longitudinal designs, independent multicenter validation, and direct comparison with established clinical models. Until these requirements are met, ncRNA profiling should be regarded as an investigational molecular approach rather than a clinically validated component of routine prediabetes assessment.

## 9. Discussion and Future Perspectives

The central question for ncRNA research in prediabetes is no longer whether circulating or tissue-associated ncRNAs differ across metabolic states, but whether they provide biologically or clinically meaningful information beyond established glycemic classification. FPG, HbA1c, and OGTT already provide practical and validated approaches for identifying prediabetes [1], and current evidence does not support replacing these measures with ncRNA-based testing. Rather, the potential value of ncRNAs lies in complementary functions that conventional glycemic measurements were not designed to capture directly, including characterization of underlying metabolic dysregulation, estimation of future progression risk [24,25], evaluation of intervention-associated molecular responses [26], and identification of regulatory pathways that may eventually provide therapeutic targets. The strength of evidence, however, differs substantially across these applications.

At the mechanistic level, miRNAs, lncRNAs, and circRNAs provide a biologically plausible framework for understanding how regulatory disturbances may emerge across skeletal muscle, liver, pancreatic β-cells, and adipose tissue during early metabolic deterioration. Representative pathways involving *H19*/let-7, *MALAT1*-associated lipogenic regulation, *MEG3*-associated β-cell regulation, CDR1as/miR-7, circHIPK3, and miR-27- and *Blnc1*-associated adipose regulation illustrate how different ncRNA classes may converge on insulin signaling, lipid metabolism, β-cell function, adipose remodeling, inflammation, and mitochondrial stress. Nevertheless, the integrated network proposed in this review should not be interpreted as a validated prediabetes-specific interactome. Much of the mechanistic evidence originates from established T2DM, obesity, insulin-resistant conditions, cellular systems, or animal models, and many proposed ceRNA relationships remain dependent on transcript abundance, binding affinity, subcellular localization, and stoichiometric requirements that have not been directly evaluated in human prediabetes [27,28,29]. The tissue-resolved framework is therefore most useful as a hypothesis-generating map for prioritizing regulatory relationships for direct testing in early dysglycemia. Among potential clinical applications, cross-sectional diagnostic discrimination currently appears less compelling than prognostic evaluation. Several circulating miRNAs can distinguish individuals with prediabetes from normoglycemic controls, but reported AUC values alone do not demonstrate that molecular testing improves upon readily available glycemic measures. By contrast, longitudinal studies directly address a clinically more relevant question: whether ncRNA profiles identify individuals with prediabetes who are more likely to progress to T2DM. The 5-year study reporting higher baseline miR-491-5p, miR-1307-3p, and miR-298 in subsequent progressors [24] and the DPP analysis of a baseline 5-miRNA score associated with incident T2DM [25] provide proof-of-concept for this application. Importantly, however, statistically significant prospective associations are not sufficient to establish clinical utility. Future prognostic models should demonstrate reproducible incremental performance beyond glucose, HbA1c, adiposity, and other established risk variables, with assessment of calibration, discrimination, reclassification, and clinical decision value.

The DPP-derived studies are particularly informative because they extend ncRNA research beyond conventional cross-sectional biomarker discovery. The baseline 5-miRNA score reported by Flowers et al. was associated with incident T2DM and also showed an exploratory treatment-by-biomarker interaction [25], whereas Lewis et al. identified longitudinal circulating miRNA changes associated with metformin exposure relative to placebo [26]. These findings represent distinct biomarker functions: the former provides prognostic evidence with exploratory predictive potential, whereas the latter provides candidate pharmacodynamic evidence. Maintaining this distinction is essential. A biomarker associated with future disease does not necessarily predict differential treatment benefit, and a molecular change occurring after treatment does not establish that the biomarker can guide treatment selection or that it mediates clinical efficacy. Clinically, these distinctions correspond to different potential decisions: a validated prognostic profile could support escalation of lifestyle support or monitoring intensity, whereas altering preventive pharmacotherapy would require a validated predictive biomarker; longitudinal pharmacodynamic signals could support response monitoring but would not by themselves guide initial treatment selection. Future prevention trials should therefore prespecify these biomarker questions and incorporate baseline and serial biospecimen collection so that prognostic, predictive, and pharmacodynamic functions can be evaluated separately within the same study.

A further possibility is that ncRNA profiling may eventually contribute to molecular phenotyping of heterogeneous prediabetes. Individuals with similar glycemic values may differ in the relative contributions of peripheral insulin resistance, hepatic metabolic dysfunction, β-cell stress, inflammation, lipid dysregulation, and mitochondrial stress. In principle, ncRNA signatures reflecting these pathways could help identify biologically distinct subgroups that are not apparent from glucose measurements alone. However, direct evidence for reproducible ncRNA-defined molecular subtypes of human prediabetes is currently insufficient. Molecular phenotyping should therefore be treated as a testable future application rather than an established use of ncRNA profiling. Future studies should determine whether integrated molecular signatures identify stable biological subgroups, whether these subgroups differ in progression risk, and ultimately whether they differ in response to preventive interventions.

Emerging small ncRNA classes may further broaden this molecular landscape. piRNAs and snoRNAs have been associated with metabolic regulation and circulating signatures in established diabetes and diabetes-related complications, although their relevance to human prediabetes remains poorly defined [30,31,32]. tRFs may be particularly relevant to early dysglycemia, as recent human islet data identified increased 5′tRF^Glu(CTC) in impaired glucose tolerance and an inverse association with insulin secretory function [33]. These findings support inclusion of broader small-RNA classes in future prediabetes profiling studies, while underscoring the need to distinguish direct human prediabetes evidence from findings in established diabetes or experimental models.

Epitranscriptomic regulation may add another dimension to ncRNA-based molecular phenotyping. m6A modification and A-to-I editing can alter RNA stability, processing, localization, and regulatory interactions and have been implicated in β-cell function, insulin resistance, adipose tissue metabolism, and nutrient-responsive signaling [35,36,37]. However, most current evidence derives from established diabetes, obesity, or experimental metabolic models, and direct profiling of these modifications in human prediabetes remains scarce. Future studies integrating ncRNA abundance with modification- or editing-specific measurements may help determine whether epitranscriptomic states provide information beyond transcript abundance alone.

Single-cell and spatial transcriptomic approaches may also help resolve one of the central limitations of circulating ncRNA studies: uncertainty regarding tissue and cellular origin. By defining cell-type-specific expression patterns and preserving spatial tissue context, these technologies could generate reference maps against which circulating ncRNA candidates can be interpreted [61]. Emerging single-cell small-RNA sequencing and spatial miRNA approaches are particularly relevant because conventional single-cell and spatial RNA-sequencing platforms incompletely capture mature miRNAs and other small ncRNAs [62,63,64]. Nevertheless, concordance between tissue-level expression and a circulating signal would not by itself establish source attribution; paired tissue–biofluid studies and orthogonal experimental validation will remain necessary.

Integration with additional molecular layers may further broaden this framework. Combining ncRNA profiles with genomics, epigenomics, proteomics, metabolomics, and detailed clinical phenotyping could provide a more comprehensive representation of the biological processes underlying early metabolic dysfunction [2,103]. Artificial intelligence and machine-learning approaches may facilitate integration of these high-dimensional datasets and identification of molecular patterns that would be difficult to detect using single-marker analyses. However, computational complexity should not be equated with clinical validity. Models derived from small or heterogeneous datasets remain vulnerable to overfitting, batch effects, population-specific signatures, and limited external generalizability. Multi-omics and AI-based models should therefore be developed using appropriately powered cohorts, independent validation datasets, transparent feature selection, and direct comparison with simpler clinical models before claims of improved precision prevention are justified.

ncRNAs also remain attractive as potential mechanistic and therapeutic targets because they can regulate multiple components of metabolic pathways. Preclinical studies have provided proof-of-concept that pharmacological modulation of miRNAs using miRNA mimics or inhibitory anti-miR/antisense approaches can alter metabolic phenotypes, including insulin resistance, lipid metabolism, inflammation, and glucose homeostasis [104,105]. In diabetes and related metabolic disorders, anti-miRNA strategies have been evaluated against multiple metabolically relevant miRNAs, although most evidence remains confined to experimental models [105]. For example, pharmacological inhibition of miR-34a in diet-induced steatohepatitis reduced hepatic lipid accumulation, inflammation, oxidative stress, and apoptosis, supporting the therapeutic potential of targeting metabolically dysregulated miRNAs [106]. Therapeutic targeting of lncRNAs and other ncRNA classes is also being explored using antisense oligonucleotides, small interfering RNAs, and related RNA-targeting strategies, but these approaches remain less mature than conventional RNA therapeutics [104]. Major barriers include achieving efficient and tissue-selective delivery, minimizing off-target effects and toxicity, controlling the magnitude and duration of target modulation, and accounting for the context-dependent and pleiotropic functions of individual ncRNAs [104,105]. These challenges are particularly important in metabolic disease, in which the same ncRNA may exert different effects across liver, skeletal muscle, adipose tissue, pancreatic β-cells, and other tissues. Moreover, molecular relationships demonstrated in established diabetes, obesity, or experimental disease models cannot be assumed to represent therapeutically actionable mechanisms during human prediabetes. Within the evidence reviewed here, no ncRNA-directed therapy has been validated for preventing progression from prediabetes to T2DM. At present, the principal therapeutic value of the ncRNA framework is therefore in target discovery and mechanistic prioritization rather than in supporting near-term ncRNA-directed treatment.

Collectively, the next phase of the field should move from candidate discovery toward staged validation. Studies should prioritize direct human prediabetes cohorts, longitudinal designs when progression is the clinical question, standardized biospecimen processing and ncRNA measurement, independent multicenter validation, and direct comparison of candidate signatures with established clinical models. Sex should likewise be treated as a prespecified biological variable rather than solely as an adjustment covariate. Adequately powered studies should test ncRNA-by-sex interactions and determine whether candidate signatures retain comparable discrimination, calibration, and clinically actionable thresholds in women and men. Importantly, variation in biospecimen processing, hemolysis control, RNA or EV isolation, analytical platform, and normalization can generate platform- or laboratory-specific ncRNA signatures, thereby limiting cross-cohort reproducibility, biomarker transferability, and the establishment of clinically applicable thresholds. Mechanistic studies should determine whether circulating signals reflect specific biological pathways without assuming tissue origin from circulating abundance alone, while intervention studies should incorporate prospective molecular sampling and prespecified biomarker functions to distinguish treatment prediction from pharmacodynamic response. Thus, the most realistic near-term contribution of ncRNA research is not a diagnostic test that replaces glucose-based classification, but a deeper molecular understanding of why individuals with apparently similar prediabetes may follow different trajectories. If future studies show that ncRNA profiles reproducibly improve progression-risk assessment, characterize biologically meaningful heterogeneity, or capture intervention responses beyond conventional measures, they could become valuable components of precision prevention. Until then, ncRNAs should be regarded primarily as mechanistic and translational research tools whose clinical utility remains to be established.

## 10. Conclusions

Prediabetes can already be identified effectively using established glycemic measures, including FPG, HbA1c, and OGTT. The potential contribution of ncRNAs therefore lies not in replacing glycemic classification, but in providing complementary information about the biological processes underlying early metabolic dysfunction. Evidence involving miRNAs, lncRNAs, and circRNAs supports regulatory roles in insulin resistance, β-cell stress, inflammation, lipid dysregulation, and mitochondrial dysfunction across metabolically relevant tissues. However, these findings derive from heterogeneous experimental and clinical settings, and the tissue-resolved ncRNA framework proposed in this review should be regarded as hypothesis-generating rather than as a validated prediabetes-specific regulatory network. Among potential translational applications, prognostic risk stratification and intervention-associated molecular monitoring currently appear more compelling than diagnostic replacement. Prospective human studies indicate that circulating miRNA profiles may be associated with future progression to T2DM. DPP-derived analyses further provide rare evidence linking baseline miRNA profiles to incident diabetes and exploratory treatment-by-biomarker interactions, while demonstrating longitudinal miRNA changes associated with metformin exposure. These findings distinguish prognostic, predictive, and pharmacodynamic biomarker functions and illustrate how ncRNA research can move beyond cross-sectional discrimination. Nevertheless, predictive treatment selection remains exploratory, ncRNA-based molecular phenotyping has not yet been validated, and therapeutic targeting of ncRNAs remains predominantly preclinical. Clinical translation will require prospective multicenter studies, standardized pre-analytical and analytical procedures, independent external validation, and direct demonstration that ncRNA profiling provides meaningful information beyond established glycemic and clinical risk factors. Future studies should therefore focus less on expanding lists of differentially expressed ncRNAs and more on validating reproducible molecular signatures linked to progression, biological heterogeneity, and intervention response. If these requirements are met, ncRNAs may ultimately contribute to a more mechanistically informed approach to precision prevention in prediabetes; at present, their strongest value lies in mechanistic discovery, hypothesis generation, and the development of candidate complementary biomarkers rather than routine clinical decision-making.

## Figures and Tables

**Table 2:** Representative intervention studies evaluating miRNA biomarkers and intervention-associated dynamics relevant to prediabetes.

Intervention	Study Design and Cohort (*n*; Context)	miRNAs	Timing/ Direction	Biomarker Interpretation	Evidence Context	Ref.
Metformin	Secondary analysis of randomized DPP cohort; adults with prediabetes; metformin *n* = 50, placebo *n* = 50; 2-year follow-up	let-7c-5p, miR-151a-3p, miR-17-5p, miR-20b-5p, miR-29b-3p, miR-93-5p	↑ after metformin vs. placebo	Candidate pharmacodynamic signature associated with metformin exposure; clinical-response tracking not established	Direct human prediabetes—randomized intervention cohort; longitudinal pharmacodynamic evidence	[26]
		miR-130b-3p, miR-22-3p, miR-222-3p, miR-320a-3p, miR-320c, miR-92a-3p	↓ after metformin vs. placebo			
DPP lifestyle, metformin, and placebo arms	Secondary analysis of randomized DPP cohort; *n* = 1000 adults with prediabetes	5-miRNA panel: miR-144, miR-186, miR-203a, miR-205, miR-206	Baseline composite score; not a post-intervention change	Prognostic: associated with incident T2DM. Exploratory predictive: treatment-by-biomarker interaction across randomized intervention groups; external validation lacking	Direct human prediabetes—randomized-trial cohort; prognostic with exploratory predictive evidence	[25]
Exercise + diet	Quasi-randomized intervention; obese male adolescents, 57 enrolled/50 completed; 10 normal-weight controls; 6 weeks; not prediabetes-specific	miR-126	↓ after intervention	Supportive intervention-associated molecular signal in an obesity-related setting; direction differs across exercise studies	Supportive human evidence—obesity-related intervention; not prediabetes-specific	[88]
Metformin	Randomized intervention in women with PCOS; *n* = 65; Rotterdam criteria; metformin, metformin + OCP, or OCP for 12 months; not prediabetes-specific	miR-122, miR-29a; miR-223	miR-122 and miR-29a: ↓ after metformin treatment; miR-223: ↓ in the metformin + OCP group	Supportive intervention-associated evidence in an insulin-resistant population	Supportive human evidence—insulin-resistant population; not prediabetes-specific	[89]
GLP-1 receptor agonist (liraglutide)	Single-arm intervention; *n* = 25 patients with T2DM; liraglutide 1.2 mg/day added to metformin for 4 months; not prediabetes-specific	miR-27b, miR-130a, miR-210	↑ after treatment	Supportive candidate pharmacodynamic evidence in T2DM; drug-specific causality not established	Supportive human evidence—single-arm T2DM intervention; not prediabetes-specific	[90]
Exercise rehabilitation	Pre/post intervention; HFpEF *n* = 60 completed + 30 healthy controls; 12 weeks; LVEF ≥ 50%, NYHA II–III; not prediabetes-specific	miR-126	↑ after rehabilitation	Supportive intervention-associated evidence from a cardiovascular population; limited relevance to prediabetes	Supportive human evidence—cardiovascular intervention; limited direct relevance to prediabetes	[91]
SGLT2 inhibitor	Experimental diabetic cardiomyopathy rat model; high-fat diet/STZ-induced diabetes	miR-30d (cardiac tissue)	↓ with SGLT2 inhibition	Preclinical mechanistic evidence; does not establish a circulating pharmacodynamic biomarker in humans	Preclinical evidence—experimental diabetic cardiomyopathy model	[92]

Evidence context describes the directness, study design, and clinical setting of intervention evidence relevant to prediabetes and does not represent a formal evidence-grading system. “Direct human prediabetes” refers to studies conducted within randomized or longitudinal prediabetes intervention cohorts, whereas “supportive human evidence” refers to intervention studies in related metabolic or cardiometabolic populations without dedicated prediabetes validation. “Preclinical evidence” denotes findings from experimental animal or cellular models. Post-intervention ncRNA changes are interpreted as candidate pharmacodynamic or intervention-associated signals and do not, by themselves, establish treatment-predictive utility or clinical efficacy. Predictive biomarker evidence requires a baseline treatment-by-biomarker interaction; accordingly, the DPP 5-miRNA score is classified as prognostic with exploratory predictive potential. None of the applications summarized here is currently validated for routine clinical use. DPP, Diabetes Prevention Program; GLP-1, glucagon-like peptide-1; HFpEF, heart failure with preserved ejection fraction; LVEF, left ventricular ejection fraction; NYHA, New York Heart Association; OCP, oral contraceptive pill; PCOS, polycystic ovary syndrome; SGLT2, sodium-glucose cotransporter 2; STZ, streptozotocin; T2DM, type 2 diabetes mellitus; ↑, increased; ↓, decreased.

## Data Availability

No new data were created or analyzed in this study. Data sharing is not applicable to this article.

## Abbreviations

The following abbreviations are used in this manuscript:

ADAR	adenosine deaminases acting on RNA
AUC	area under the receiver operating characteristic curve
BAT	brown adipose tissue
ceRNA	competing endogenous RNA
circRNA	circular RNA
DPP	Diabetes Prevention Program
EV	extracellular vesicle
FPG	fasting plasma glucose
GLP-1RA	glucagon-like peptide-1 receptor agonist
HbA1c	glycated hemoglobin
IFG	impaired fasting glucose
IGT	impaired glucose tolerance
lncRNA	long non-coding RNA
m6A	N6-methyladenosine
miRNA	microRNA
MISEV	Minimal Information for Studies of Extracellular Vesicles
ncRNA	non-coding RNA
NGT	normal glucose tolerance
OGTT	oral glucose tolerance test
PCOS	polycystic ovary syndrome
piRNA	PIWI-interacting RNA
RT-qPCR	reverse transcription quantitative polymerase chain reaction
SGLT2	sodium-glucose cotransporter 2
snoRNA	small nucleolar RNA
T2DM	type 2 diabetes mellitus
tRFs	tRNA-derived fragments
WAT	white adipose tissue
